# Growth improvement of wheat (*Triticum aestivum*) and zinc biofortification using potent zinc-solubilizing bacteria

**DOI:** 10.3389/fpls.2023.1140454

**Published:** 2023-05-12

**Authors:** Murad Ali, Iftikhar Ahmed, Hamza Tariq, Saira Abbas, Munir Hussain Zia, Amer Mumtaz, Muhammad Sharif

**Affiliations:** ^1^ National Culture Collection of Pakistan (NCCP), Land Resources Research Institute (LRRI), National Agricultural Research Centre (NARC), Islamabad, Pakistan; ^2^ Department of Soil and Environmental Sciences, The University of Agriculture, Peshawar, Pakistan; ^3^ Cereal Crops Research Institute (CCRI), Pirsabak, Nowshera, Pakistan; ^4^ Department of Zoology, University of Science and Technology, Bannu, Pakistan; ^5^ Research and Development Department, Fauji Fertilizer Company (FFC) Limited, Rawalpindi, Pakistan; ^6^ Food Sciences Research Institute (FSRI), National Agricultural Research Centre (NARC), Islamabad, Pakistan

**Keywords:** Zn solubilizing bacteria, PGPR - plant growth-promoting rhizobacteria, IAA, P-solubilisation, nifH and acdS genes, wheat

## Abstract

Zinc (Zn) is an indispensable element for proper plant growth. A sizeable proportion of the inorganic Zn that is added to soil undergoes a transformation into an insoluble form. Zinc-solubilizing bacteria (ZSB) have the potential to transform the insoluble Zn into plant-accessible forms and are thus promising alternatives for Zn supplementation. The current research was aimed at investigating the Zn solubilization potential of indigenous bacterial strains and to evaluate their impact on wheat growth and Zn biofortification. A number of experiments were conducted at the National Agriculture Research Center (NARC), Islamabad, during 2020-21. A total of 69 strains were assessed for their Zn-solubilizing ability against two insoluble Zn sources (ZnO and ZnCO_3_) using plate assay techniques. During the qualitative assay, the solubilization index and solubilization efficiency were measured. The qualitatively selected Zn-solubilizing bacterial strains were further tested quantitatively using broth culture for Zn and phosphorus (P) solubility. Tricalcium phosphate was used as insoluble source of P. The results showed that broth culture pH was negatively correlated with Zn solubilization, i.e., ZnO (r^2 =^ 0.88) and ZnCO_3_ (r^2 =^ 0.96). Ten novel promising strains, i.e., *Pantoea* sp. NCCP-525, *Klebsiella* sp. NCCP-607, *Brevibacterium* sp. NCCP-622, *Klebsiella* sp. NCCP-623, *Acinetobacter* sp. NCCP-644, *Alcaligenes* sp. NCCP-650, *Citrobacter* sp. NCCP-668, *Exiguobacterium* sp. NCCP-673, *Raoultella* sp. NCCP-675, and *Acinetobacter* sp. NCCP-680, were selected from the ecology of Pakistan for further experimentation on wheat crop based on plant growth-promoting rhizobacteria (PGPR) traits, i.e., solubilization of Zn and P in addition to being positive for *nif*H and *acd*S genes. Before evaluating the bacterial strains for plant growth potential, a control experiment was also conducted to determine the highest critical Zn level from ZnO to wheat growth using different Zn levels (0.1, 0.05, 0.01, 0.005, and 0.001% Zn) against two wheat varieties (Wadaan-17 and Zincol-16) in sand culture under glasshouse conditions. Zinc-free Hoagland nutrients solution was used to irrigate the wheat plants. As a result, 50 mg kg^-1^ of Zn from ZnO was identified as the highest critical level for wheat growth. Using the critical level (50 mg kg^-1^ of Zn), the selected ZSB strains were inoculated alone and in consortium to the seed of wheat, with and without the use of ZnO, in sterilized sand culture. The ZSB inoculation in consortium without ZnO resulted in improved shoot length (14%), shoot fresh weight (34%), and shoot dry weight (37%); with ZnO root length (116%), it saw root fresh weight (435%), root dry weight (435%), and Zn content in the shoot (1177%) as compared to the control. Wadaan-17 performed better on growth attributes, while Zincol-16 had 5% more shoot Zn concentration. The present study concluded that the selected bacterial strains show the potential to act as ZSB and are highly efficient bio-inoculants to combat Zn deficiency, and the inoculation of these strains in consortium performed better in terms of growth and Zn solubility for wheat as compared to individual inoculation. The study further concluded that 50 mg kg^-1^ Zn from ZnO had no negative impact on wheat growth; however, higher concentrations hampered wheat growth.

## Introduction

Agriculture has remained Pakistan’s most important industry, accounting for one-fifth of the country’s overall gross domestic product (GDP), despite the country’s rapid growth in industrial and technological sectors. In Pakistan, wheat is widely regarded as one of the most important staple crops (Mirza et al., 2015). Most Pakistanis are accustomed to consuming wheat (*Triticum aestivum* L) three times daily in the form of pancakes called “chapati” (Ahsin et al., 2020). Agricultural practices are increasingly focusing on novel techniques that address both productivity and micronutrient deficiency (hidden hunger). Most cereal grains have micronutrient deficiencies, which impact over 2 billion people worldwide (Waqeel and Khan, 2022). Living creatures need Zn in modest levels for healthy growth and development (Huang et al., 2022). It contributes to the metabolism of carbohydrates and auxin in plants (Hussain et al., 2019), and it is also a powerful antioxidant (Singh et al., 2018). Plants with a Zn shortage have slower development, chlorosis, and smaller leaves (Zafar et al., 2018). Similarly, wheat with Zn deficiency has pale leaves and stunted growth. Those who consume diets low in Zn can become insufficient in Zn. Furthermore, contact with high Zn levels has a noxious effect, though it is very rare. Symptoms of Zn toxicity in plants typically appear when Zn leaf concentrations are beyond 300 mg kg^-1^ on a dry weight basis, though toxicity thresholds can vary greatly even within the same species (Broadley et al., 2007). It is reported that Zn deficiency is a severe and the fourth major micronutrient deficiency, which affects the health of approximately 66 percent of humans globally (Zhang et al., 2012). In Pakistan, 19% of children (<5 year) in the population suffers greatly from low Zn dietary intake (Gop and Unicef, 2019). The main reasons of Zn deficiency in humans are due to the improper nutritional intake of Zn. The average male needs 8.94 milligrams of Zn per day, whereas the average female needs only 7.58 milligrams of Zn per day; however, these numbers can vary greatly depending on age and diet (Wang et al., 2022).

The majority of agricultural land is Zn inadequate or contains Zn in unavailable forms to crop. It has been reported by the Food and Agriculture Organization (FAO) that 50% of the world’s agricultural soil is Zn deficient. It has been reported that in Pakistan 70% of cultivated land is Zn deficient (Imtiaz et al., 2010). Crop production has been negatively affected, and the nutritional quality of the food produced has suffered as a result of Zn shortage in agricultural soils, leading to major nutritional and health problems. However, Zn scarcity issues in crops are related to poor Zn solubility in soils rather than low total Zn levels (Rassaei et al., 2020). In fact, soils have high total Zn content, which is mostly in fixed forms and not available to plants. The pH and moisture content of the soil influence Zn solubility (Athokpam et al., 2018). Zinc deficiency arises because of Zn deficiency in the soil, and it is one of the most common micronutrient deficiencies. Zinc fertilizers in the form of Zn sulphate or Zn-EDTA (Anuradha et al., 2015) have been applied, but they are ineffective in the long run, since 96.0–99.0% of the applied Zn is transformed into unavailable Zn pools by precipitation to carbonates, oxides, or phosphates, etc. (Zhang et al., 2017). Other strategies include traditional breeding, transgenic procedures, and genetic engineering to combat Zn deficiency (Khokhar et al., 2020; Wang K. et al., 2020). However, these technologies are expensive, time-consuming, and slower. Zinc-solubilizing rhizobacteria provide a better alternative to all these approaches. In this context, using Zn-solubilizing plant growth-promoting rhizobacteria as inoculants is a cost-effective and environmentally benign option for Zn biofertilization. Several different PGPR have been discovered to be efficient Zn solubilizers. These bacteria boost plant growth and development by colonizing the rhizosphere and solubilizing complicated Zn compounds into simpler ones. This makes Zn more readily available to the plants, which in turn boosts plant growth and development (Singh and Prasanna, 2020). Microorganisms that are capable of solubilizing Zn do so through a number of different methods, one of which is acidification. Bacteria in the soil produce organic acids, lowering the pH and sequestering Zn cations (Rasul et al., 2019). In addition, Zn can be made more soluble *via* chelation with the anions (Kamran et al., 2017). One such process is the production of siderophores that could be implicated in Zn solubilization (Ajmal et al., 2021). Plants with multiple PGPR inoculations have shown improved growth and Zn content. These include *Bacillus aryabhattai* (Prathap et al., 2022), *Bacillus subtilis* (Samaras et al., 2022), and *Pseudomonas* and *Rhizobium* (Naz et al., 2016). Among the bacterial strains to exhibit Zn solubilization on a laboratory scale are *Pseudomonas* spp., (Hashemnejad et al., 2021), *Klebsiella* sp., *Acinetobacter* sp., *Gluconacetobacter* sp.*, Burkholderia* sp., *Serratia* sp., (Haroon et al., 2022), *Citrobacter* sp., and *Enterobacter* sp., (Ajmal et al., 2021). Gandhi et al. (2022) confirmed that root length of wheat seedlings increases by 16-40% through the inoculation of microbes. The inoculation of a bacterial strain with the intention of boosting the Zn nutrient availability for plants is a significant practice that is required in agriculture. Keeping in view the importance of the Zn and soil micro-organism, the present investigations were carried out with the hypothesis that potential ZSB may help in alleviating Zn deficiency in plants and enhancing growth and Zn accumulation in wheat. To test this hypothesis, a series of experiments were conducted on a selection of promising bacterial strains, which solubilize the insoluble Zn sources, and the shortlisted ZSB strains were tested for multifarious abilities, including P solubilization and molecular characterization. The selected most potent ZSB strains were also evaluated on their growth promotion and Zn solubility for wheat plants in sand culture under control conditions.

## Materials and methods

To assess the bacteria for Zn solubility, 69 previously isolated bacteria were grown from the glycerol stocks of the “National Culture Collection of Pakistan (NCCP)”, Islamabad, Pakistan. The strains, with detailed information such as their I.D, accession numbers, nucleotide length (bp), closely related taxa, and similarity percentages, are presented in **Supplementary Table 1**.

### Assessment of Zn-solubilizing potential through plate assay

The bacterial strains obtained were assessed for Zn solubility using a Bunt and Rovira agar medium ((NH4)_2_SO_4_ 1 g; glucose 10 g; KCl 0.2 g; K_2_HPO_4_ 0.1 g; MgSO_4_ 0.2 g; agar 15 g L^-1^) comprising 0.1% Zn in the form of ZnCO_3_ and ZnO (Bunt and Rovira, 1955). The inoculated plates were kept in an incubator at 30°C for eight days.

The halo zones formed by the strains around the bacteria colony showed Zn solubilization potential, which was assessed by the halo zone diameter. We used the following formula stated by Gontia-Mishra et al. (2017) to determine the solubilization index (SI) and solubilization efficiency (SE):


$$SI=\frac{Diameter\;of\;colony+Diameter\;of\;halozone}{Diameter\;of\;colony}$$

$$SE=\frac{So\mathrm{lub}ilization\;diameter}{Colony\;growth\;diameter}\times 100$$

### Quantification of Zn solubilization using broth assay

The Zn solubilization potential of the bacterial strains that showed positive activity on the plate assay was also quantified. For this purpose, the overnight grown bacterial culture was inoculated into the Bunt and Rovira broth (glucose 10 g; (NH4)_2_SO_4_ 1 g; KCl 0.2 g; K_2_HPO_4_ 0.1 g; MgSO_4_ 0.2 g L^-1^) containing 0.1% Zn from ZnO and ZnCO_3_ (Bunt and Rovira, 1955). The media added with Zn that had no bacterial strain were served as the control. All tubes were incubated at 30°C for 6 days at 180rpm in an IS-RDD3 incubator shaker. After the incubation period, the culture broth was passed through a 0.45 µm pore size Nylon syringe. The clear supernatant was collected, and the soluble concentration of Zn in the supernatant was analyzed using the Varian SpectrAA 220FS atomic absorption spectrophotometer at the wavelength of 213.9 nm. The quantity of solubilized Zn by bacteria was expressed as microgram mL^-1^ (µg Zn mL^-1^). The experiment was conducted in a completely randomized (CR) design in three replicates (Fasim et al., 2002; Sharma et al., 2011).

### Characterization of potent Zn-solubilizing strains

#### Phosphorus solubilization ability

The Zn-solubilizing bacterial strains were further screened for phosphorus solubilization on National Botanical Research Institute Phosphate (NBRIP) agar media (Glucose 10 g, Ca_3_ (PO_4_)_2_ 5 g, MgCl_2_.6H_2_O 0.25 g, KCl 0.2 g, (NH_4_)_2_SO_4_ 0.1 g agar 15 g L^-1^ and adjusted pH 7.0) (Nautiyal, 1999; Aliyat et al., 2020). During the qualitative screening, each ZSB strain was inoculated on the NBRIP agar media petri plates and kept at 30°C for eight days. Around the colony, the strains produced a clear zone that showed P solubilization, which was measured from the clear zone diameter according to the recommended method of Gontia-Mishra et al. (2017). The most efficient phosphate solubilized strains on the plate assay were quantified in the NBRIP broth. The overnight grown bacterial culture was inoculated into the NBRIP broth at the rate of 0.75 mL into each test tube. The media without bacterial strain was served as the control. All inoculated test tubes were incubated at 30°C for 6 days. After the shaking period, a clear supernatant was obtained in the falcon tubes. Ammonium bicarbonate-diethylenetriaminepentaacetic acid (AB-DTPA) solution was added into a 5 mL supernatant (Soltanpour and Workman, 1981). The mixture was shaken on the reciprocating shaker in an open flask. The ascorbic acid method was used for broth P. One mL supernatant containing AB-DTPA solution was taken into a 50 mL conical flask, into which was added 9 mL of distilled water and 2.5 mL of freshly prepared color mixed reagent. After 15 minutes of incubation in the dark, a blue color developed in the mixture, which was then quantified by a spectrophotometer at 880 nm to determine the concentration of accessible P (Watanabe and Olsen, 1965). The experiment was performed in triplicate.

#### Compatibility test

The selected bacterial strains for the pot experiment were evaluated for their growth compatibility according to the procedure of Raja et al. (2006), also mentioned by Sohaib et al. (2020). In different combinations, the strains were streaked perpendicularly on tryptone soya agar media. The cross-streaked agar plates were photographed to illustrate the colony line and inhibition zone at the intersection of the strains after being incubated at 30°C for 72 hours.

#### Molecular characterization

The genomic deoxyribonucleic acid (DNA) of the ZSB strain was extracted according to the cetyltrimethylammonium bromide (CTAB) protocol, with certain modifications (Wilson, 1987). The *nifH* gene and the *acdS* gene were used to characterize the Zn-solubilizing bacterial strains at the molecular level for plant growth-promoting activities and the Zn-solubilizing potential. The most effective Zn-solubilizing bacterial strains with PGPR activity based on the *nifH* and *acdS* genes were selected for further plant experimentation.

### Amplification of *nifH* and *acdS* genes

The presence of the *acdS* and *nifH* genes in the bacterial strains selected for their capacity to dissolve Zn was examined. Three diverse sets of primers (nifHF/nifHI, PolF/PolR, and nifHfor/nifHrev) were selected for amplification of the *nifH* gene (Laguerre et al., 2001; Poly et al., 2001; Sarita et al., 2008). The total (20 µL) reaction mixture contained template DNA (5 µL), the reverse and forward primers each having 1.5 µL, Premix ExTaq (10 µL) (Takara, Japan), and polymerase chain reaction (PCR) water (10 µL). In the same way, for the amplification of the *acdS* gene, three set of primers, i.e., F1936f/F1938r, F1936f/F1939r, and F1937f/F1938r, were used as described by Blaha et al. (2006). Gel electrophoresis in a 0.8% agarose gel was used to examine the amplification products.

#### A: Standardization of highest critical Zn level for wheat growth in sand culture

A control experiment was conducted to determine the highest critical Zn level for wheat growth and the Zn concentration in shoots prior to evaluating the solubilization capacity of Zn-solubilizing bacterial strains in wheat plants. The experiment was carried out in plastic pouches/bags filled with sand under glasshouse conditions at the Bio-resource Conservation Institute (BCI), National Agriculture Research Center (NARC), Islamabad, during the rabi season of 2020-21 to assess the effect of various levels of Zn form insoluble ZnO. The plastic pouch (length 19 cm and diameter 10.5 cm) was filled with 800 g sieved sand. The insoluble Zn source of ZnO was thoroughly mixed with sand from each plastic pouch. Each plastic pouch was punctured at the bottom and placed in pots/trays, and four seeds of wheat were sown in them. The treatment (Control (No Zn), 0.1, 0.05, 0.01, 0.005, and 0.001% Zn) was studied on two commercially available wheat varieties (Wadaan-17 and Zincol-16). There were three replications of each treatment, and the experiment was arranged in a completely randomized design. Zinc-free Hoagland nutrient solution was used to irrigate the plants (Hoagland, 1933), as mentioned by De Bever et al. (2012). After five weeks, the plants were harvested, and the data were recorded on root length, shoot length, and Zn concentration in the shoot and root according to standard procedure, as mentioned by Gupta et al. (2021) and Kamran et al. (2017).

#### B: Effect of identified Zn-solubilizing bacterial strains on wheat growth and Zn content in shoot

To evaluate the inoculation effect of the chosen Zn-solubilizing bacterial strains on wheat growth and shoot Zn content, a plant experiment was conducted at the glass house of NARC, Islamabad, during the rabi season of 2020-21. The plastic pouches/bags were filled with 800 g of sterilized sieved sand. Insoluble Zn source ZnO, at the rate of 0.005% (50 mg kg^-1^) Zn, was added to each pouch and uniformly mixed with sand. The plastic pouches were punctured at the bottom and placed in pots/trays. The following 26 treatment combinations were studied on the wheat varieties.

Treatment combination details

**Table:** 

S.NO	Treatment combinations	S.NO	Treatment combinations
1.	Control (No Zn and strain)	14.	Selected bacterial strains in consortium
2.	ZnO	15.	ZnO + *Pantoea* sp. NCCP-525
3.	*Pantoea* sp. NCCP-525	16.	ZnO + *Klebsiella* sp. NCCP-607
4.	*Klebsiella* sp. NCCP-607	17.	ZnO + *Brevibacterium* sp. NCCP-622
5.	*Brevibacterium* sp. NCCP-622	18.	ZnO + *Klebsiella* sp. NCCP-623
6.	*Klebsiella* sp. NCCP-623	19.	ZnO + *Acinetobacter* sp. NCCP-644
7.	*Acinetobacter* sp. NCCP-644	20.	ZnO + *Alcaligenes* sp. NCCP-650
8.	*Alcaligenes* sp. NCCP-650	21.	ZnO + *Citrobacter* sp. NCCP-668
9.	*Citrobacter* sp. NCCP-668	22.	ZnO + *Exiguobacterium* sp. NCCP-673
10.	*Exiguobacterium* sp. NCCP-673	23.	ZnO + *Raoultella* sp. NCCP-675
11.	*Raoultella* sp. NCCP-675	24.	ZnO + *Acinetobacter* sp. NCCP-680
12.	*Acinetobacter* sp. NCCP-680	25.	ZnO + *Pseudomonas* sp. NCCP-436
13.	*Pseudomonas* sp. NCCP-436	26.	ZnO + bacterial strains in consortium

### Seed collection and disinfection

The seeds of commercially available wheat varieties (Wadaan-17 and Zincol-16) were surface-sterilized with 70% ethanol followed by 3% sodium hypochlorite (NaOCl) solution for 1 min and then washed with sterilized distilled water (Jain et al., 2020). The Zn-solubilizing bacterial strains were grown in TS broth in a 500 mL Erlenmeyer flask on an incubator shaker (120 rpm) at 30°C for 24 hours. The seeds were carefully transferred to sterilized petri plates, and the respective strains were inoculated to the wheat seeds and placed overnight. Using sterile forceps, the overnight healthy wheat seeds were transferred into the pouches, and 1 mL of bacterial inoculum was given to each respective plastic pouch. Zinc-free Hoagland nutrient solution was used to irrigate the plants.

### Experimental design and data collection

The pots were arranged in a completely randomized design. The experiment was conducted at three replications and were harvested five weeks after sowing, and the plants roots and shoots were separated after being washed with tap water. The length and fresh and dry weight of both the roots and shoots were measured.

After harvesting, the sample was dried in an oven at 70°C for five days to evaluate the Zn concentration in the wheat seedlings. A 0.2 g ground sample was weighed and digested with a mixed acid (HNO_3_: HClO_4 =_ 3:1, v/v). On a hot plate, the mixture was heated up until it turned clear. Whatman filter paper was used to filter the digested dry matter content, which was then diluted in 50 mL of distilled water and examined with an atomic absorption spectrophotometer to determine the amount of total Zn (Wei et al., 2022).

### Statistical analysis

The collected data were statistically analyzed according to the experimental design using Statistix 8.1 software. To compare the significant difference between the treatment means, the least significant difference test was applied at a 5% level of probability (Steel and Torrie, 1980).

## Results

### Assessment of Zn-solubilizing potential through plate assay

The qualitative solubilization of insoluble Zn (ZnO and ZnCO_3_) by the ZSB bacterial strains is shown in **Table 1**. In the initial screening, 24 bacteria tested positive for ZnO and 21 for ZnCO_3_, as reported by Ali et al. (2022) (**Supplementary Table 2**). The zinc oxide-supplemented medium had a substantially higher zone of solubilization than the ZnCO_3_ medium. The results obtained from the *in vitro* screening of selected strains for their ability to solubilize ZnO revealed that these strains solubilized Zn ranging from 2.6 to 4.3 SI and from 164 to 327% SE after 8 days of incubation.

**Table 1:** Solubilization index and solubilization efficiency of bacterial strains during plate assay using insoluble source of zinc and phosphorus.

Zinc-solubilizing strains	ZnO		ZnCO_3_		Ca_3_(PO_4_)_2_	
	SI	SE (%)	SI	SE (%)	SI	SE (%)
*Bacillus* sp. NCCP-49	2.80 i*	181.8 h*	–	–	–	–
*Lysinibacillus* sp. NCCP-54	3.26 h	227.8 g	–	–	3.69 de*	269.54 de*
*Brevundimonas* sp. NCCP-147	3.2 h	224.1 g	3.02 h	202.1 h	2.92 j	192.83 j
*Klebsiella* sp. NCCP-195	3.33 h	232.7 g	2.71 j	170.7 j	–	–
*Serratia* sp. NCCP-200	3.53 g	252.9 f	3.006 h	200.56 h	–	–
*Pantoea* sp. NCCP-241	4 def	300.8 de	–	–	3.28 h	227.94 h
*Sphingobacterium* sp. NCCP-246	2.63 j	164 i	2.34 m	134.5 m	–	–
*Pantoea* sp. NCCP-525	4 def	303.8 de	3.49 e	249.1 e	3.60 f	260.07 f
*Citrobacter* sp. NCCP-605	4 def	300.6 de	3.42 f	242.2 f	3.30 h	230.04 h
*Klebsiella* sp. NCCP-607	4.2 abc	319.7 abc	3.6 f	260.2 d	3.66 ef	265.66 ef
*Alcaligenes* sp. NCCP-616	2.86 i	185.5 h	3.12 g	212.4 g	–	–
*Brevibacterium* sp. NCCP-622	3.93 f	294.2 e	3.77 b	277.7 b	3.62 f	262.46 f
*Klebsiella* sp. NCCP-623	4.1 bcde	310 bcd	3.69 c	269.5 c	3.75 d	275.19 d
*Staphylococcus* sp. NCCP-628	2.93 i	194.4 h	2.34 m	134.5 m	–	–
*Klebsiella* sp. NCCP-631	3.23 h	224.1 g	2.91 i	191 i	–	–
*Acinetobacter* sp. NCCP-644	4 ef	295.9 e	3.62 d	262.6 d	3.45 g	245.05 g
*Pseudomonas* sp. NCCP-646	2.9 i	188.3 h	2.58 l	158.4 l	–	–
*Alcaligenes* sp. NCCP-650	3.7 g	266.4 f	3.08 g	208.6 g	2.66 k	165.88 k
*Pseudomonas* sp. NCCP-654	3.2 h	221.2 g	2.64 k	164.8 k	3.07 i	206.83 i
*Citrobacter* sp. NCCP-668	4.2 ab	321.3 ab	3.71 c	270.9 c	3.30 h	230.04 h
*Exiguobacterium* sp. NCCP-673	4.1 bcd	311.9 bcd	3.82 a	282.1 a	3.83 c	283.76 c
*Raoultella* sp. NCCP-675	4.3 a	327.6 a	3.59 d	259.2 d	3.91 b	291.16 b
*Acinetobacter* sp. NCCP-680	4.1 cdef	307.6 cde	3.81 ab	281.2 ab	4.00 a	300 a
*Brachybacterium* sp. NCCP-936	3.2 h	221 g	2.69 j	169.3 j	–	–
**LSD _(0.05)_**	0.14	13.56	0.045	4.18	0.067	6.66

(-) = the sources are not solubilized; SI, solubilization index; SE, solubilization efficiency; NCCP, National Culture Collection of Pakistan.

*Mean values followed by same letter (s) are not significantly different at the P ≤ 0.05.

The zinc-solubilizing strains *Citrobacter* sp. NCCP-668, *Raoultella* sp. NCCP-675, and *Exiguobacterium* sp. NCCP-673 were statistically at par with *Acinetobacter* sp. NCCP-680 and *Klebsiella* sp. NCCP-623, which showed considerable maximum SI values of 4.3, 4.2, 4.1, 4.1, and 4.1, respectively, and SE values of 327, 321, 311.9, 310, and 307.6%, respectively. The lowest SI values of 2.6 and 2.8 and SE values of 164 and 181% were obtained with inoculation of the bacterial strain of *Sphingobacterium* sp. NCCP-246 and *Bacillus* sp. NCCP-49 for ZnO, respectively. Similarly, in terms of ZnCO_3_, the utmost SI value of 3.82 and SE value of 282% were observed by the NCCP-673 and statistically equivalent with NCCP-680 isolated from industrial effluent. It was followed by the *Brevibacterium* sp. NCCP-622 and NCCP-623. The petri plates inoculated with *Staphylococcus* sp. NCCP-628 had the lowest SI value of 2.34 and SE value of 134.5%, making them statistically equivalent with the plates containing the NCCP-246 bacterial strain.

### Quantification of Zn-solubilizing bacteria potential

**Figure 1:**
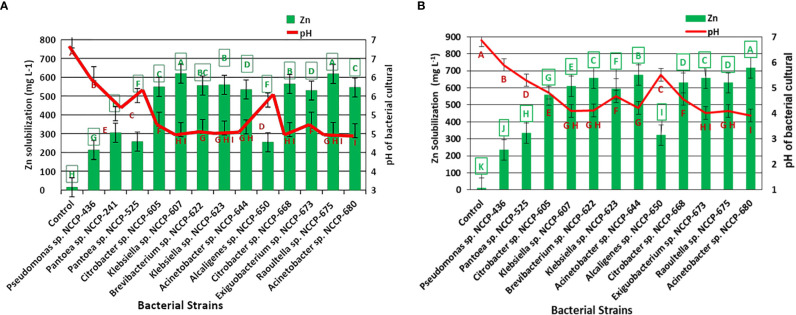
Quantification of Zn solubilized by Zn-solubilizing bacterial strains from **(A)** ZnO and **(B)** ZnCO_3_ and pH of broth medium. Alphabets above and below the error bars represent the significant difference among datasets for Zn and pH, respectively.

Based on the highest values of SI and SE in the plate assay supplemented with ZnO and ZnCO_3_, 12 (*Pantoea* sp. NCCP-241, *Pantoea* sp. NCCP-525, *Citrobacter* sp. NCCP-605, *Klebsiella* sp. NCCP-607, *Brevibacterium* sp. NCCP-622, *Klebsiella* sp. NCCP-623, *Acinetobacter* sp. NCCP-644, *Alcaligenes* sp. NCCP-650, *Citrobacter* sp. NCCP-668, *Exiguobacterium* sp. NCCP-673, *Raoultella* sp. NCCP-675, and *Acinetobacter* sp. NCCP-680), and 11 (NCCP-525, NCCP-605, NCCP-607, NCCP-622, NCCP-623, NCCP-644, NCCP-650, NCCP-668, NCCP-673, NCCP-675, and NCCP-680) bacterial strains were selected, respectively. For the quantitative Zn solubilization, the selected strains were inoculated into broth supplemented with ZnO and ZnCO_3_. The results demonstrated that every bacterial strain under study had the potential to dissolve Zn in the broth. However, the efficacy of the strains depended on the Zn source (ZnCO_3_ and ZnO), with ZnCO_3_ being solubilized by the strains more effectively than ZnO (**Figures 1A, B**). It was revealed from the finding of the quantitative Zn solubilization that the utmost solubilized concentration of insoluble ZnO was shown by the bacterial strains *Klebsiella* sp. NCCP-607 (61.9%) and *Raoultella* sp. NCCP-675 (61.7%), followed by the strain NCCP-668, which were statistically equivalent to NCCP-623 and NCCP-622. These strains were able to dissolve 304 into 619 mg L^-1^ of ZnO. The mean minimum quantity of solubilized Zn (14 mg L^-1^) and (213 mg L^-1^) were recorded from the control (no inoculation of bacterial strain) and with the inoculation of the bacterial strain *Pseudomonas* sp. NCCP-436, which was used as an insoluble bacterial strain, respectively (**Figure 1A**).

**Figure 2:**
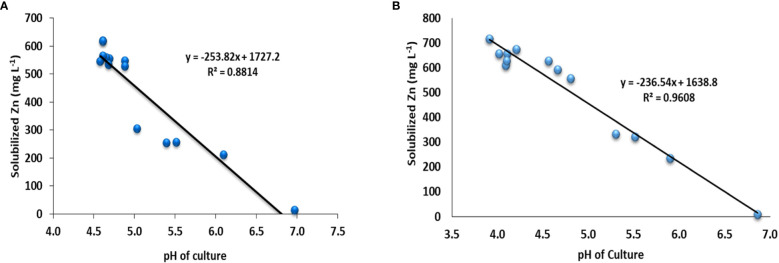
Correlation of broth pH and Zn concentration from insoluble **(A)** ZnO and **(B)** ZnCO_3_.

The quantitative Zn solubilization results obtained in the broth amended with ZnCO_3_ revealed that the maximum Zn (718 mg L^-1^) was solubilized by the bacterial strain NCCP-680, followed by *Acinetobacter* sp. NCCP-644, while the minimum Zn solubilized (10 mg L^-1^) and (235 mg L^-1^) in broth, which was not inoculated to bacterial strain (control) and was inoculated with *Pseudomonas* sp. NCCP-436, respectively (**Figure 1B**). Furthermore, the pH of the broth culture was negatively correlated with Zn solubilization from the insoluble Zn source, i.e., ZnO (r^2 =^ 0.88, **Figure 2A**) and ZnCO_3_ (r^2 =^ 0.96, **Figure 2B**). A significant (P ≤ 0.05) decline in the broth pH was recorded by the inoculation of the ZSB as compared to the non-inoculated broth (control) (**Figure 1**). The inoculation of the bacterial strains NCCP-680, NCCP-675, NCCP-668, NCCP-623, and NCCP-607 resulted in the greatest pH decrease (4.6) of the liquid medium amended with ZnO, whereas the broth inoculated with NCCP-436 caused the least pH decrease (6.1). Similarly, in the ZnCO_3_-amended medium, the pH value of 7 ± 0.2 significantly decreased to a range from 3.9 to 5.9. The broth of NCCP-680 had the lowest pH value of 3.9, followed by strain NCCP-673, and presented higher acidity due to bacterial growth, while highest pH value of 5.9 was recorded from the broth inoculated with NCCP-436.

### Characterization of Zn-solubilizing strains

#### Phosphate solubilization

The Zn-solubilizing bacterial strains were screened for P solubilization. Out of the 24 ZSB strains, only 15 were able to solubilize phosphorus in mineral salts when tri-calcium phosphate was added to the agar medium (**Table 1**). The qualitative screening of the ZSB strains-solubilized phosphate ranged from 2.66 to 4.0 SI and from 165.8% to 300% SE (**Table 1**). The strain NCCP-680 solubilized the maximum P (4.0 and 300% SI and SE, respectively) from tri-calcium phosphate, followed by NCCP-675. The strains NCCP-673 and NCCP-623 also showed a better response for phosphate solubilization. The mean minimum phosphorus solubilization index (PSI) of 2.66 and phosphorus solubilization efficiency (PSE) of 165.8% were observed by the inoculation of the bacterial strain *Alcaligenes* sp. NCCP-650. Based on the maximum value of PSI and PSE, 12 bacterial strains (*Pantoea* sp. NCCP-241, *Pantoea* sp. NCCP-525, *Citrobacter* sp. NCCP-605, *Citrobacter* sp. NCCP-668, *Klebsiella* sp. NCCP-607, *Klebsiella* sp. NCCP-623, *Brevibacterium* sp. NCCP-622, *Acinetobacter* sp. NCCP-644, *Acinetobacter* sp. NCCP-680, *Alcaligenes* sp. NCCP-650, *Exiguobacterium* sp. NCCP-673, and *Raoultella* sp. NCCP-675) were selected. For the quantification of phosphate solubility, the selected strains were inoculated into broth and amended with tri-calcium phosphate.

**Figure 3:**
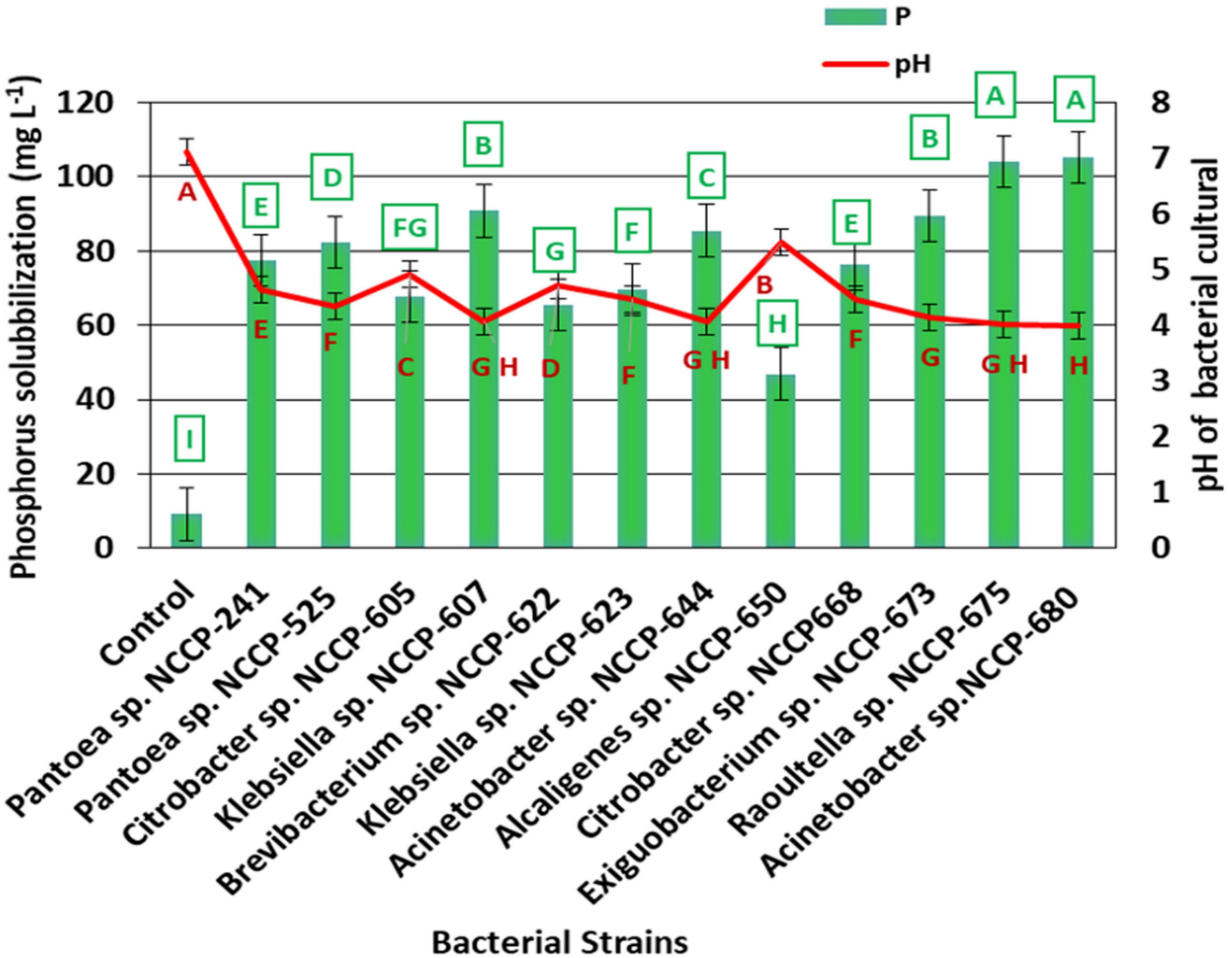
Effect of zinc-solubilizing bacterial strains on phosphorus solubilization and pH of broth. Alphabets **(A–I)** above and below the error bars represent the significant difference among datasets for phosphorus and pH, respectively.

**Figure 4:**
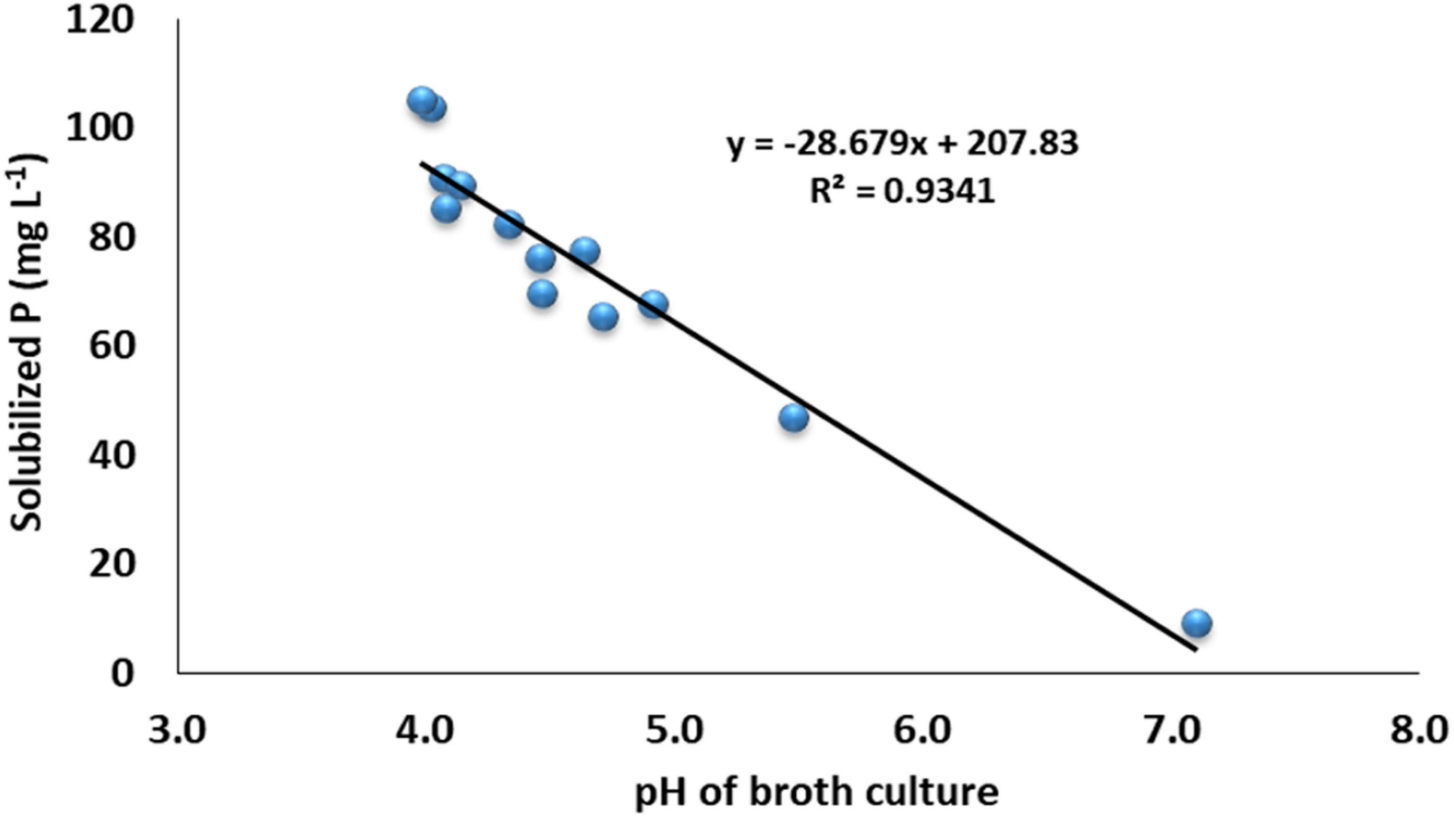
Correlation of broth pH and phosphorus concentration from insoluble Ca_3_ (PO_4_)_2_.

The data revealed that the ZSB strains exhibited significant differences in the solubilization of P and ranged from 9.1 to 105.2 mg L^-1^. The maximum phosphate solubilization of 105.2 mg L^-1^ was observed from the inoculation of bacterial strain NCCP-680, which was statistically at par with NCCP-675, followed by the strains NCCP-607 and NCCP-673, as they solubilized phosphate of 90.2 and 89.5 mg L^-1^, respectively (**Figure 3**). The mean minimum solubilization of 9 mg L^-1^ was recoded from the control. The data regarding the broth pH revealed that inoculation of the strains decreased the broth pH and solubilized the insoluble P. The decline in the broth pH was recorded with the inoculation of NCCP-680 and NCCP-675, as they dropped the pH value by up to 4.0, followed by the strains NCCP-673, NCCP-644, and NCCP-607 (**Figure 3**). Moreover, the pH of the broth culture was negatively correlated with P solubilization (r^2 =^ 0.93, **Figure 4**).

#### Compatibility among bacterial strains

**Figure 5:**
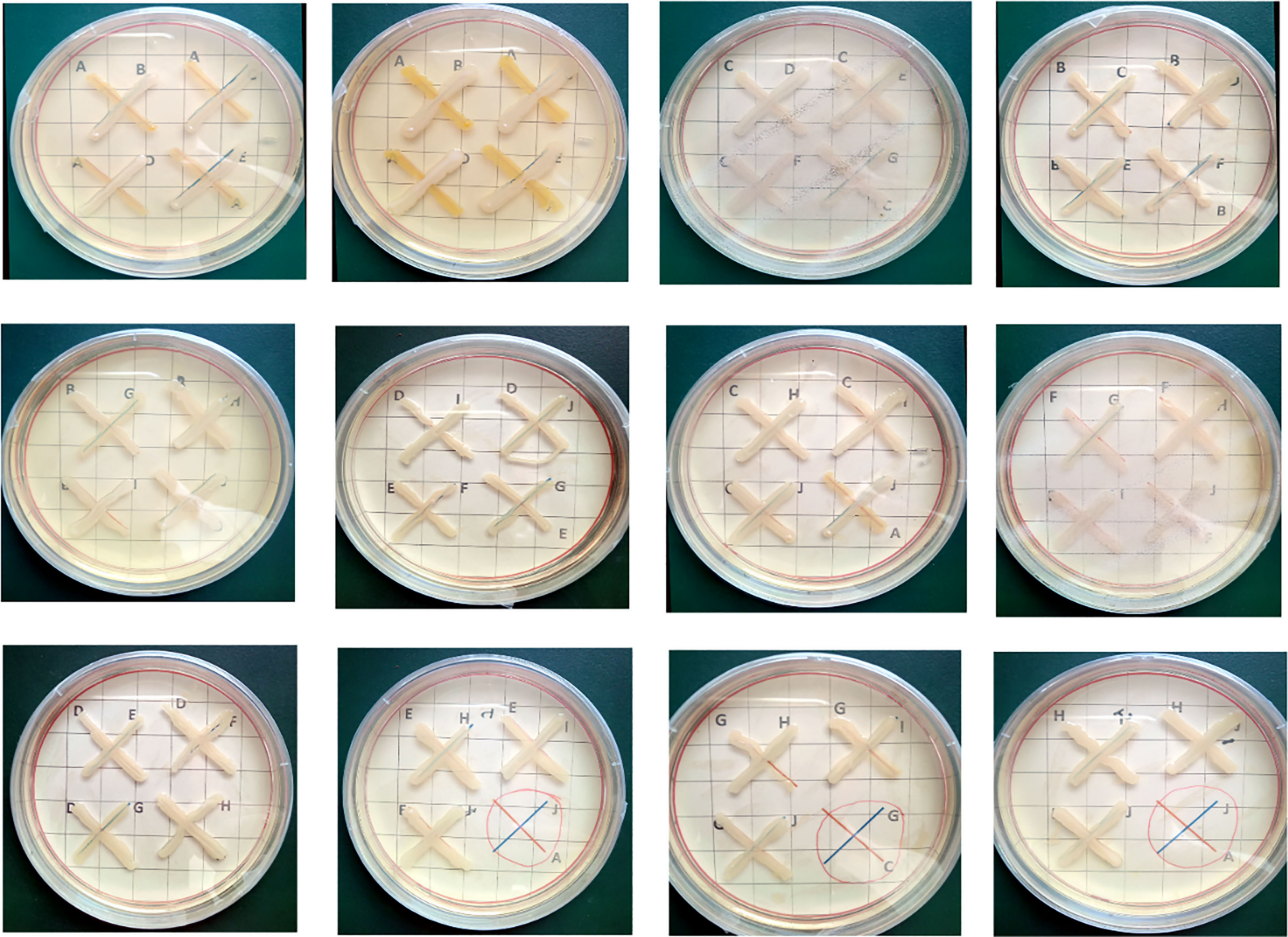
Cross-streak test between each strain of compatible combinations. Each of the zinc- solubilizing strains was streaked perpendicularly in the order shown using arrows. Alphabets **(A-J)** within each plate represent different bacterial strains. **^A^** NCCP-525; **^B^** NCCP-607; **^C^** NCCP-622; **^D^** NCCP-623; **^E^** NCCP-644; **^F^** NCCP-650; **^G^** NCCP-668; **^H^** NCCP-673; **^I^** NCCP-675; **^J^** NCCP-680.

The 10 potent Zn-solubilizing bacteria (NCCP-525, NCCP-607, NCCP-622, NCCP-623, NCCP-644, NCCP-650, NCCP-668, NCCP-673, NCCP-675, and NCCP-680) were examined for their growth compatibility. The strains showed no growth inhibition after 72 hours of incubation (**Figure 5**).

### Molecular characterization using PCR

#### Amplification of *nifH* gene

Nitrogenase enzymes are essential for reducing nitrogen to ammonia and are controlled by the *nifH* gene. The existence of the *nifH* gene in Zn-solubilizing bacteria is an indication of their plant growth-promoting ability. The selected Zn-solubilizing bacterial strains (NCCP-525, NCCP-607, NCCP-622, NCCP-623, NCCP-644, NCCP-650, NCCP-668, NCCP-673, NCCP-675, and NCCP-680) and *Rhizobium etli* (JCM 21823^T^) were used as the control. To confirm the *nifH* gene in the selected ZSB through PCR, three diverse sets of primers were used (**Table 2**). The results demonstrated that the *nifH* gene was confirmed in six different strains, i.e., *Brevibacterium* sp. NCCP-622, *Klebsiella* sp. NCCP-607, *Klebsiella* sp. NCCP-623, *Alcaligenes* sp. NCCP-650, *Raoultella* sp. NCCP-675, and *Acinetobacter* sp. NCCP-680, using the nifHF/nifHI primer set. Meanwhile, no bands were observed for the DNA of other bacterial strains (**Table 2**). Similarly, the strains NCCP-525, NCCP-650, and NCCP-675 showed positive results when using the primer set of PolF/PolR (**Table 2**). The primer set nifHfor/nifHrev, which amplified the *nifH* gene in the DNA, was isolated from NCCP-525, NCCP-607, NCCP-622, NCCP-623, NCCP-650, and NCCP-680 (**Table 2**). Moreover, it was confirmed from the amplification of the *nifH* gene that all strains have an N-fixing ability, except for NCCP-644, NCCP-668, and NCCP-673, which showed negative results from all three sets of primers (**Table 2**).

**Table 2:** Confirmation of *nifH* and *acdS* gene in zinc-solubilizing bacteria by using different sets of primers.

S. No	Name of the strains	*nifH* gene (nifHF/nifHI)	*nifH* gene (PolF/PolR)	*nifH* gene (nifHfor/nifHrev)	*acdS* gene (F1936f/F1938r)	*acdS* gene (F1936/F1939r)	*acdS* gene (F1937f/F1939r)
1.	*Pantoea* sp. NCCP-525	–	+	+	–	–	–
2.	*Klebsiella* sp. NCCP-607	+m	–	+m	+m	+m	+m
3.	*Brevibacterium* sp. NCCP-622	+m	–	+m	+m	+m	+m
4.	*Klebsiella* sp. NCCP-623	+m	–	+	+m	+m	+m
5.	*Acinetobacter* sp. NCCP-644	–	–	–	+m	–	–
6.	*Alcaligenes* sp. NCCP-650	+m	+	+	–	+m	+m
7.	*Citrobacter* sp. NCCP- 668	–	–	–	–	–	–
8.	*Exiguobacterium* sp. NCCP- 673	–	–	–	+m	–	–
9.	*Raoultella* sp. NCCP- 675	+m	+	–	+m	+m	+m
10.	*Acinetobacter* sp. NCCP-680	+m	–	+m	+	+m	+m
11.	*Rhizobium etli* (JCM 21823^T^)	+	+	+	–	+	+

+, PCR of the probable size; -, no PCR product; +m, expected PCR products plus other products of unpredicted and non-specific size.

#### Amplification of *acdS* gene

The enzyme 1-aminocyclopropane-1-carboxylate deaminase is responsible for degrading 1-aminocyclopropane-1-carboxylate and producing ammonia and α-ketoglutarate, which enhance plant growth, and this enzyme is encoded by the *acdS* gene. To amplify the *acdS* gene in the selected ZSB, three sets of primers (F1936/F1938, F1936/F1939, and F1937/F1938) were used. A total of 10 strains were screened for the *acdS* gene, and 7 strains (NCCP-607, NCCP-622, NCCP-623, NCCP-644, NCCP-673, NCCP-675, and NCCP-680) showed positive results using the primer F1936f/F1938r (**Table 2**). Meanwhile, the primer F1936/F1939r showed bands in six strains (NCCP-607, NCCP-622, NCCP-623, NCCP-650, NCCP-675, and NCCP-680) (**Table 2**). Similarly, the primer F1937f/F1939r amplified the *acdS* gene in strains NCCP- 607, NCCP-622, NCCP-623, NCCP-650, NCCP-675, and NCCP-680 (**Table 2**). The strains NCCP- 607, NCCP-622, NCCP-623, and NCCP-675 showed positive results, while NCCP-525 and NCCP-668 showed negative results with the three sets of primers.

Based on Zn and the phosphorus solubilization from their respective ores and the presence of nifH and acdS genes, the 10 bacterial strains (NCCP-525, NCCP-607, NCCP-622, NCCP-623, NCCP-644, NCCP-650, NCCP-673, NCCP-675, and NCCP-680) were designed as potential Zn-solubilizing bacteria and selected for a control experiment under sand culture on growth and Zn solubility for wheat from ZnO.

#### A: Standardization of highest critical Zn level for wheat growth in sand culture

**Growth parameters:** Statistical analyses of the data in **Table 3** revealed that the Zn levels and wheat varieties significantly (P ≤ 0.05) affected the shoot length and root length. The results illustrated that as the level of Zn increased, the shoot length decreased, while the root length increased. The mean maximum shoot length (46 cm) was recorded from the pouches that were applied 0.001% Zn from ZnO and statistically at par with the plastic pouches that were applied 0.005% Zn, and this was followed by the control pouch. The pouches that received 0.1% Zn produced a mean minimum shoot length of 26.1 cm. In terms of varieties, Wadaan-17 achieved 10% more shoot length than Zincol-16.

**Table 3:** Effect of Zn from zinc oxide on wheat growth and shoot Zn concentration.

Treatment (T) (% Zn)	Shoot length (cm)	Root length (cm)	Shoot Zn Content (mg kg^-1^)	Root Zn Content (mg kg^-1^)
control (No Zn)	43.9 b*	17 d*	48 f	110 f
0.1	26.1 e	21.2 a	906 a	1124 a
0.05	30.2 d	20.7 ab	845.8 b	1015 b
0.01	37 c	20.3 b	728 c	906 c
0.005	45.6 a	19.2 c	273 d	506 d
0.001	46 a	17.7 d	95.8 e	202 e
LSD_0.05_	1.19	0.92	33.5	25.5
Varieties (V)				
Wadaan-17	40 a	19 b	472 b	675 a
Zincol-16	36.3 b	19.6 a	493.6 a	613 b
LSD_0.05_	0.68	0.53	19.3	14.7
Interaction effect				
V×T	**Figure 6A**	NS	NS	**Figure 6B**

NS, non-significant; * Means of the same category followed by different letters are significantly different at 5% level of probability.

**Figure 6:**
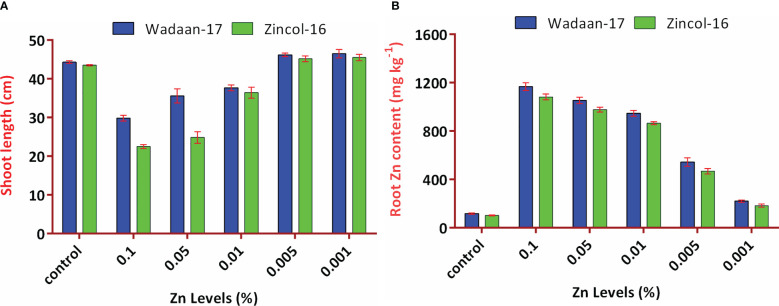
Interaction effect of Zn levels from ZnO and wheat varieties on **(A)** shoot length and **(B)** root Zn content. Error bars represent the standard error.

The root length showed different trends, with increasing levels of Zn from ZnO. The mean maximum root length (21.2 cm) was recorded from the pouches that received 0.1% Zn and statistically at par with the plastic pouches that received 0.05% Zn. The control pouch produced a minimum root length of 17 cm and was statistically equivalent to the pouches that received 0.001% Zn. In terms of varieties, Zincol-16 had 3.1% more root length than Wadaan-17. The recorded interaction effect of the Zn levels and wheat varieties was highly significant for the shoot length (**Figure 6A**) but non-significant (P ≥ 0.05) for the root length.

**Zn content in shoot and root:** The data regarding the shoot and root Zn content in wheat showed that there were highly significant (P ≤ 0.01) variances among the treatments and wheat varieties (**Table 3**). The applied Zn level form ZnO enhanced the shoot and root Zn content, which ranged from 48 to 906 mg kg^-1^ and 110 to 1124 mg kg^-1^, respectively. The mean minimum shoot and root Zn content of 48 and 110 mg kg^-1^ were recorded in the control pouch, respectively. The mean maximum shoot and root Zn content of 906 and 1124 mg kg^-1^ were recorded in pouches treated with 0.1% Zn, respectively. The finding showed that as the Zn level from ZnO increased, the shoot and root Zn content enhanced toxicity and significantly stunted (P ≤ 0.01) shoot growth. In terms of varieties, Zincol-16 had 4.5% more shoot Zn content than Wadaan-17, while 10% more Zn content was recorded in the roots of Wadaan-17 than Zincol-16. The interactive effect of varieties and treatments was found to be statistically non-significant for the shoot Zn content but significant for the root Zn content, as shown in **Figure 6B**.

#### B: Effect of identified Zn-solubilizing bacterial strains on wheat growth and Zn content in shoot

Based on the results of the laboratory study, 10 potent Zn solubilizing bacterial strains were selected for the subsequent glasshouse experiment under sand culture on wheat. The selected Zn-solubilizing bacterial strains were inoculated alone and in combination with Zn from the insoluble Zn source (ZnO). Statistical analyses of the data regarding the growth attributes and Zn concentration in the shoot revealed a highly significant difference among the treatment combinations and wheat varieties for the growth and Zn concentration in the shoot. The interaction effect of treatment combinations and varieties was to be found significant (**Table 4**).

**Table 4:** Influence of Zn-solubilizing bacteria on growth attributes and shoot Zn concentration of wheat varieties under sand culture.

Treatments (T)	Shoot length (cm)	Root length (cm)	Shoot fresh weight (g/plant)	Root fresh weight (g/plant)	Shoot dry weight (g/plant)	Root dry weight (g/plant)	Shoot Zn Concentration (mg kg^-1^)
Control	40.8 cd*	15.9 f*	1.45 f*	0.121 i*	0.496 e*	0.039 h*	27.7 k*
Insoluble Zn	46.1 ab	23 cd	2.11 a	0.282 f	0.827 a	0.074 ef	240 f
NCCP-525	43.5 abc	20 de	1.5 f	0.157 ghi	0.520 de	0.05 gh	31.5 ijk
NCCP-607	45.1 ab	20.5 de	1.6 f	0.163 ghi	0.560 d	0.053 fgh	34.9 hij
NCCP-622	45.4 ab	21 d	1.61 ef	0.164 ghi	0.559 d	0.052 gh	39.9 gh
NCCP-623	45 ab	20.5 de	1.61 ef	0.166 ghi	0.560 d	0.054 fgh	35.7 hij
NCCP-644	45 ab	20.6 de	1.61 ef	0.156 ghi	0.561 d	0.049 gh	40 gh
NCCP-650	44 abc	19 def	1.57 f	0.146 ghi	0.548 de	0.047 gh	38.6 gh
NCCP-668	45 ab	20 de	1.76 de	0.172 ghi	0.621 c	0.055 fgh	40 gh
NCCP-673	45.3 ab	21.8 d	1.77 d	0.166 ghi	0.630 bc	0.053 fgh	38 ghi
NCCP-675	45.3 ab	20.7 de	1.78 d	0.180 gh	0.632 bc	0.055 fgh	40 gh
NCCP-680	45.7 ab	21 d	1.8 cd	0.177 gh	0.640 bc	0.057 fgh	40.4 gh
NCCP-436	42 bc	16.6 ef	1.45 f	0.127 hi	0.497 e	0.04 h	30 jk
Microbe in consortium	46.5 a	22.8 d	1.95 bc	0.199 g	0.683 b	0.065 efg	43 g
ZnO + NCCP-525	36 ef	27 bc	0.93 g	0.481 e	0.334 f	0.156 cd	290 d
ZnO + NCCP-607	35.4 ef	28 b	0.94 g	0.558 bc	0.330 f	0.172 bcd	303.6 c
ZnO + NCCP-622	36.3 ef	28.6 b	0.91 g	0.564 bc	0.321 f	0.172 bcd	312 b
ZnO + NCCP-623	34.5 ef	28.5 b	0.93 g	0.541 bcd	0.325 f	0.166 bcd	315.6 b
ZnO + NCCP-644	36 ef	28.9 b	0.91 g	0.535 cde	0.321 f	0.164 bcd	312 b
ZnO + NCCP-650	36.3 ef	28.5 b	0.91 g	0.495 de	0.319 f	0.153 d	295 d
ZnO + NCCP-668	34.6 ef	29 b	0.91 g	0.572 bc	0.322 f	0.175 bc	318 b
ZnO + NCCP-673	37 de	29 b	0.95 g	0.592 b	0.334 f	0.182 b	312 b
ZnO + NCCP-675	35.7 ef	30.5 ab	0.94 g	0.550 bcd	0.329 f	0.171 bcd	317 b
ZnO + NCCP-680	36 ef	30.7 ab	0.95 g	0.585 bc	0.337 f	0.184 b	318 b
ZnO + NCCP-436	44.9 abc	22 d	2.08 ab	0.291 f	0.845 a	0.081 e	248 e
ZnO + consortium	32.5 f	34.5 a	0.88 g	0.648 a	0.348 f	0.209 a	354 a
LSD_0.05_	4	4.1	0.16	0.054	0.059	0.022	6.7
Wheat Varieties (V)							
Wadaan-17	45.7 a*	22.7 b*	1.73 a*	0.425 a*	0.633 a*	0.131 a*	165.6 b*
Zincol-16	35.9 b	25.7 a	1.02 b	0.251 b	0.351 b	0.079 b	174 a
LSD_0.05_	0.35	0.35	0.014	0.004	0.005	0.0019	1.86
Interaction effect							
V×T	**Figure 7A**.	**Figure 7B**.	**Figure 7C**.	**Figure 7D**.	**Figure 7E**.	**Figure 7F**.	**Figure 7G**.

**NCCP-525**= Pantoea sp.; **NCCP-607**= Klebsiella sp.; **NCCP-622**= Brevibacterium sp.; **NCCP-623**= Klebsiella sp.; **NCCP-644**= Acinetobacter sp.;

**NCCP-650**= Alcaligenes sp.; **NCCP-668**= Citrobacter sp.; **NCCP-673**= Exiguobacterium sp.; **NCCP-675**= Raoultella sp.;

**NCCP-680**= Acinetobacter sp.; **NCCP-436**= Pseudomonas sp.; consortium= combination of selected Zn-solubilizing bacteria.

*Means of the same category followed by different letters are significantly different at 5% level of probability.

**Growth parameters:** Statistical analyses of the data concerning the shoot length, root length, shoot fresh weight, root fresh weight, shoot dry weight, and root dry weight of wheat plants exhibited enormously significant (P ≤ 0.01) variations among the treatment combinations and varieties (**Table 4**). The results showed that Zn-solubilizing bacteria have a positive effect on the shoot length, root length, shoot fresh weight, root fresh weight, shoot dry weight, and root dry weight, while the shoot length, shoot fresh weight, and shoot dry weight significantly were impaired with ZSB inoculated in combination with zinc oxide.

The plastic pouches that were inoculated with bacterial strains in consortium produced a maximum shoot length of 46.5 cm, which was statistically equivalent to that of those pouches treated with ZSB strains and ZnO alone and *Pseudomonas* sp. NCCP-436+ZnO. Similarly, a maximum shoot fresh weight (2.11g plant^-1^) and shoot dry weight (0.827g plant^-1^) were recorded in pouches treated with ZnO alone, which were statistically at par with the pouches treated with NCCP-436+ZnO. The pouch that received bacterial strains in consortium+ZnO produced a minimum shoot length of 32.5 cm, shoot fresh weight of 0.88g, and dry shoot weight of 0.348, making it statistically equivalent with the pouch that received ZSB alone with ZnO. These results depict that the root length, root fresh weight, and root dry weight were significantly improved in the pouch treated with consortium ZSB+ZnO.

The pouches that were inoculated with consortium ZSB+ZnO produced a maximum root length (34.5 cm), root fresh weight (0.648 g), and root dry weight (0.209 g), making them statistically at par with the pouch that received ZSB alone with ZnO. The control pouches a produced minimum root length (15.9 cm), root fresh weight (0.121 g), and root dry weight (0.039 g).

**Figure 7:**
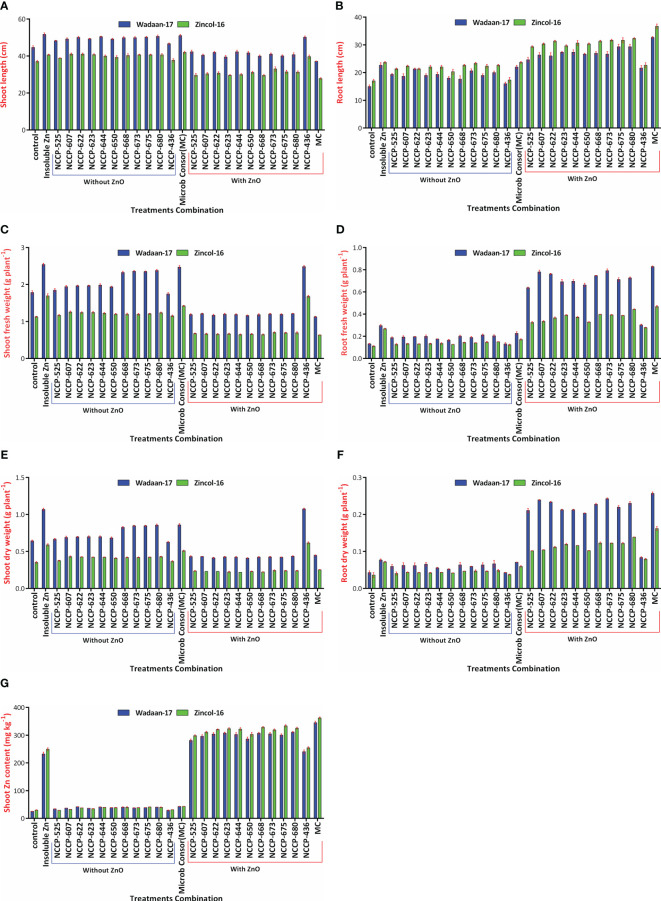
Interaction effect of varieties and treatments on **(A)** shoot length, **(B)** root length, **(C)** shoot fresh weight, **(D)** root fresh weight, **(E)** shoot dry weight, **(F)** root dry weight, and **(G)** shoot Zn content.

Furthermore, as for the wheat varieties, Wadaan-17 had achieved a greater shoot length (27%), shoot fresh weight (69%), root fresh weight (69%), shoot dry weigh (80%), and root dry weight (65.8%) than Zincol-16. Meanwhile, Zincol-16 had produced 13% more root length than Wadaan-17. The interactive result of the varieties (V) and treatment combination (T) was statistically significant for the shoot length (**Figure 7A**), root length (**Figure 7B**), shoot fresh weight (**Figure 7C**), root fresh weight (**Figure 7D**), shoot dry weight (**Figure 7E**), and root dry weight (**Figure 7F**).

**Zn content in shoot:** Statistical analyses revealed that there were highly significant (P ≤ 0.01) variations among the treatment combinations and varieties for the shoot Zn content (**Table 4**). The plastic pouch inoculated with the ZSB strain enhanced Zn content in the shoot more than the control pouch and ranged from 27.7 to 354 mg kg^-1^. The control pouch had the lowest shoot Zn content of 27.7 mg kg^-1^. The highest shoot Zn content of 354 mg Kg^-1^ was recorded in the pouches that were treated with consortium ZSB+ZnO. The pouches treated with ZnO alone enhanced Zn content in the shoot more than the control pouches, and this was associated with the ability of plants to utilize the available portion of Zn from ZnO; they were statistically at par with the pouches that received *Pseudomonas* sp. NCCP-436+ZnO. The results further demonstrated that the pouches inoculated with consortium ZSB+ZnO solubilized the Zn from insoluble ZnO and enhanced the Zn concentration of the shoot (723%) toxicity and resulted in extremely stunted shoot growth of 43% more than the pouches that received bacterial strains in consortium without ZnO. In terms of varieties, Zincol-16 had 5% more shoot Zn content than Wadaan-17. The interaction effect of V×T was found to be significant for the shoot Zn content (**Figure 7G**). The 10 selected potent novel species of Zn-solubilizing bacteria in consortium had a prominent effect on plant growth and enhanced Zn solubilization under sand culture in glasshouse conditions.

## Discussion

Zinc is an indispensable micronutrient that makes a significant contribution to physiological and metabolic processes in plants and humans (Natasha et al., 2022). Worldwide, particularly in developing countries, Zn is one of the most commonly deficient nutrients in plants and humans due to crops being grown on Zn-deficient soils (Zia et al., 2020). Zn fertilizers applied to the soil plants can only use a low quantity because of the unavailability due to alkaline and the calcareous nature of soils (Mumtaz et al., 2017). Different methods are now employed to eradicate wheat Zn deficiency. Utilizing Zn-solubilizing plant growth-promoting rhizobacteria is one of the most cost-effective and environmentally beneficial methods.

These bacteria are inoculated into the soil, where they produce chelating agents, extrude organic acids, and reduce soil Zn deficiency (Masood et al., 2022), which finally fortify the part of the cereal that could be eaten (Ramesh et al., 2014; Krithika and Balachandar, 2016). We qualitatively and quantitatively evaluated 69 bacterial strains for this aim. The plate assay findings demonstrated that Zn-solubilizing bacteria had a high potential to solubilize insoluble Zn sources such as ZnO and ZnCO_3_. The outcomes of the current research are congruent with the conclusions of Bhakat et al. (2021) and Prathap et al. (2022), who examined the potential for Zn solubilization in various bacterial strains.

The bacteria utilized in this investigation displayed varying degrees of halo zone around the colony, denoted as a sign of solubilization; the solubilization variations from the same insoluble source may be caused by bacteria belonging to various genera. The outcomes showed that among the insoluble Zn sources (ZnCO_3_ and ZnO), the bacteria solubilized ZnO more than ZnCO_3_ on the plate assay (**Table 1**). Other research has also reported variations in the solubilization of ZnO and ZnCO_3_. According to Hina et al. (2018) and Dinesh et al. (2018), in a plate assay, bacteria inoculated to ZnO formed a maximum halo zone compared to ZnCO_3_. In the current study, the bacterial strains showed a variable degree of solubilization, and the findings of the present experiment are supported by Sunithakumari et al. (2016) and Dinesh et al. (2018). Among the tested bacterial strains, *Citrobacter* sp. NCCP-668, *Raoultella* sp. NCCP-675*, Acinetobacter* sp. NCCP-680*, Klebsiella* sp. NCCP-623, and *Exiguobacterium* sp. NCCP-673 showed the highest solubilization, and similar results were reported by Gandhi and Muralidharan (2016) and Wang J. et al. (2020). Due to some limitations, the plate assay technique is not considered reliable to evaluate the solubilization ability of bacterial strains. Therefore, the potential bacterial strains from the plate assay were selected to be tested in broth/quantitatively as supplemented with the insoluble Zn source (ZnO and ZnCO_3_). In the quantification assay, the tested bacterial strains performed well, and the results showed that more Zn was solubilized than control (un-inoculated) and inoculated with *Pseudomonas* sp. NCCP-436 (insoluble bacterial strain) (**Figure 1**); these results are corroborated in the previous report by Hina et al. (2018). Similarly, in broth, the tested strains had a variable response to Zn solubilization from ZnO and ZnCO_3_. The maximum Zn was solubilized from ZnCO_3_ by the bacterial strains, and it might be possible that theses strains are better adapted to the calcareous nature of Pakistan’s soil conditions; these findings are in line with the results of Hina et al. (2018), who reported that irrespective of bacterial strains, more Zn was solubilized from insoluble ZnCO_3_ in the Bunt and Rovira broth medium. The other possible reason for ZnCO_3_ solubilization by the bacterial strains could be the higher affiliation of tested bacterial strains with carbonate particles. The solubilization of Zn from insoluble compounds by the Zn-solubilizing bacteria might be due to the production of different organic acids. Moreover, in the present experiment, the pH of the inoculated Zn-solubilizing strains culture was negatively co-related with solubilized Zn in broth (**Figure 2**). This might be a result of the production of organic acid, and similar results were also documented by Hina et al. (2018) and Dinesh et al. (2018), who noted that the Zn-solubilized bacterial strain reduced the broth pH. Several scientists have reported that Zn-solubilizing bacteria produce various types of organic acids, such as lactic acid, gluconic acid, and oxalic acid, and consequently reduce the pH and solubilize the insoluble source of Zn (Abaid-Ullah et al., 2015; Masood et al., 2022).

In the current experiment, the selected Zn-solubilizing strains were evaluated for further plant growth promotion activities, such as P solubilization and molecular characterization. In terms of phosphate solubilization, it was recorded that Zn-solubilizing strains exhibited phosphate solubilization both qualitatively and quantitatively. Qualitative phosphate solubilization was noticed with the presence of the halo zone. Meanwhile, the phosphate solubilization by the strains was quantified in broth. The potential bacterial strains were selected in a qualitative assay for quantification in broth. In addition, the bacterial strains, which showed a potential response for Zn solubilization, also outperformed for phosphate solubilization in both the qualitative and quantitative assays, and a similar finding was recorded by Naseer et al. (2020), who documented that Zn-solubilizing *Bacillus* strains also solubilized the phosphate. The results of present study are consistent with the conclusion of Aliyat et al. (2020); Raths (2019), and Yang and Yang (2020), who reported different species of *Acinetobacter*, *Raoultella*, and *Klebsiella* for the solubilization of phosphate. The solubilization of phosphate owes to the significant production of various organic acids or to enhancing the chelation of the cations fully bound to phosphorus. The present study reported a strong negative co-relation between P solubilization and the broth culture pH (**Figure 4**). The present results are in conformity with the results of Chauhan et al. (2017), who indicated a negative correlation between the pH and P solubilization from an insoluble source.

Nitrogen is one of the essential macro-nutrients (Yin et al., 2018), and the PGPR bacterial strains are capable of fixing atmospheric nitrogen to ammonia *via* direct strategies (Gouda et al., 2018). Kim and Rees (1994) reported that the gene responsible for nitrogen fixation, named as *nifH*, is present in both free-living and symbiotic bacteria. Chauhan et al. (2017) described that the *nifH* gene is present in many bacteria beside rhizobia, and a similar finding was also elaborated by Abaid-Ullah et al. (2015), who reported the presence of the *nifH* gene in different bacterial strains. In the present study, three types of primers were used for amplification of the *nifH* gene. Among the ten bacterial strains, seven showed expression of the *nifH* gene (**Table 2**). The bacterial strains showing the *nifH* gene included *Pantoea* sp. NCCP-525*, Klebsiella* sp. NCCP-607, *Klebsiella* sp. NCCP-623*, Brevibacterium* sp. NCCP-622*, Alcaligenes* sp. NCCP-650*, Raoultella* sp. NCC-675, and *Acinetobacter* sp. NCCP-680, as has been earlier reported by Abbas (2016) and Singh et al. (2021). It is documented that bacterial enzymes such as 1-aminocyclopropane-1-carboxylate (ACC) deaminase alleviate drought stress by lowering the level of plant hormone ethylene, which deaminates its precursor ACC into α-keto butyrate and ammonia (Govindasamy et al., 2008). In current experiment, amplification of the *acdS* genes through PCR showed that eight Zn-solubilizing bacterial strains were positive for ACC deaminase activity (**Table 2**). Moreover, all the positive strains the PCR yielded non-specific bands along with the bands of expected size, and similarly, non-specific bands were also observed by Govindasamy et al. (2008) during the amplification of the *acdS* genes in the DNA of bacteria isolated from wheat rhizosphere. Therefore, the present Zn-solubilizing bacterial strains can tolerate stress and help plants to withstand in a stress environment. Our results are supported by Singh et al. (2021), who amplified the *acdS* genes in different strains isolated from the rhizosphere of sugarcane. Recently, different bacterial genera were identified by several scientists that have the ability of ACC deaminase activity, i.e., *Achromobacter, Brevibacterium, Alcaligenes*, *Pantoea, Azospirillum*, and *Klebsiella* (Kang et al., 2010; Abbas, 2016; Singh et al., 2021). Moreover, most of the strains used in this experiment are not reported for Zn solubilization and for plant growth-promoting activities. However, some plant growth-promoting abilities, i.e., phosphorus solubilization, N-fixation, IAA, and organic acid production potential, were recorded by Raths (2019). The current findings are further corroborated by Malviya et al. (2012), who claimed that *Acinetobacter* could be exploited as a well-known plant growth-promoting rhizobacteria, and Vaid et al. (2013), who further described the bacteria for IAA production and Zn solubilization.

### Standardization of highest critical Zn level for wheat growth in sand culture

**Figure 8:**
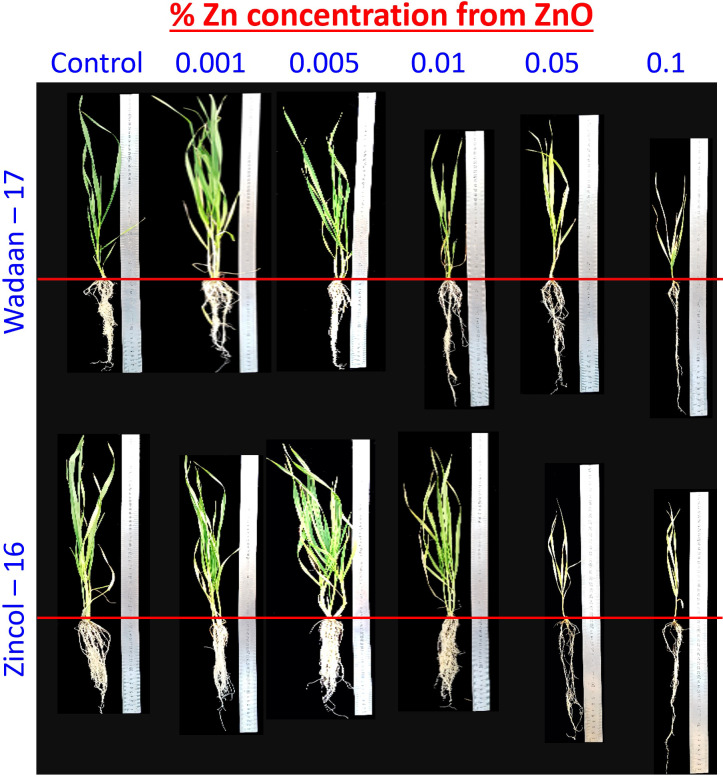
Effect of zinc concentration from zinc oxide on growth of wheat varieties under sand culture.

**Growth parameters:** Plant growth requires Zn, and when it is present both in low and high amounts, it can stunt plant growth due to deficiency and toxicity, respectively (Natasha et al., 2022). Before evaluating the Zn solubilization potential of the selected bacteria, we first have to determine the threshold levels of Zn from zinc oxide to wheat growth. Yang et al. (2011) and Caldelas et al. (2012) reported that growth parameters are the best indicator under stress, and their response can be seen when plant gets exposed to a high concentration of heavy metal. The results of our experiment showed that the Zn level above 0.005% significantly decreased the shoot length and enhanced the root length (**Table 3** and **Figure 8**). The visible symptoms of the dwarf wheat seedling, the damaged pigment content, and slower development of leaf were observed in the current experiment. In regard to the application of Zn levels to wheat seedling, the current findings are corroborated by those of Glińska et al. (2016). The current results demonstrated that pouches treated with an increased Zn concentration saw the shoot length decreased significantly. The impaired shoot length might be due to the inhibition of meristematic cell division and elongation of the roots cell. Jain et al. (2010) observed an 88% cell division inhibition of *Saccharum* sp. at a Zn concentration of 130 mg L^-1^. However, in terms of the root length, the findings of the current investigation are in contrast with those of Glińska et al. (2016), who recorded the depletion of the root length of wheat grown hydroponically for 7 days at a Zn concentration of 300 mg L^-1^. However, in the present experiment, the enhanced root length might be due to the metabolic activities of Zn in the plant roots.

**Zn content in shoot and root:** On a dry weight basis, a plant usually contains Zn in the range of 10 to 100 mg kg^-1^, and toxicity appears if the Zn concentration becomes greater than 300 mg kg^-1^ (Broadley et al., 2007). The results of present study showed that by increasing the Zn level, the shoot and root Zn content increased significantly (**Table 3**). The Zn content in the control can be correlated with the presence of Zn in sand culture. Application of the Zn dose of 0.001% and 0.005% from ZnO to wheat under sand culture did not deplete the shoot length, and no toxicity symptoms were observed in the plant. The findings of current investigation are congruent with those of the study by Glińska et al. (2016), who demonstrated that increasing the applied Zn concentration disturbed the ion balance in wheat plant. The present finding revealed that a Zn application greater than 0.005% from ZnO impaired the growth of wheat plant, and the plant showed toxicity; the results are dissimilar from the finding of Kamran et al. (2017), who incorporated 0.5% Zn from ZnCO_3_ to wheat but did not observe a decline in the growth parameter and toxicity symptoms. The most possible reason could be the sensitivity to Zn within the same species, as reported by Jin et al. (2008), who recorded that a high Zn accumulating ecotype of Sedum alfredi performed well when Zn was applied up to 500 μM, while toxic effects were recorded in a non-Zn hyper-accumulating ecotype of Sedum alfredi when 50 μM Zn was applied. The results showed that the maximum Zn content is more present in the plant roots than the shoot. The probable reason of these behaviors might be due to the binding of Zn to opposite charged sites in the cell walls of the plant root or due to the enhancement of Zn storage in the vacuole of the cell and as a result of the reduced Zn translocation to the shoot at the presence of higher Zn availability (Greger, 2004). Zincol-16 transports greater Zn from the root to shoot, as similarly observed by Imtiaz et al. (2017), who recorded the efficient and inefficient wheat genotypes on the basis of Zn translocation under field condition. To the best of our knowledge, the present study is the first report to standardize the highest level of Zn from ZnO under sand culture for wheat growth.

### Effect of identified Zn-solubilizing bacterial strains on wheat growth and Zn content in shoot

**Figure 9:**
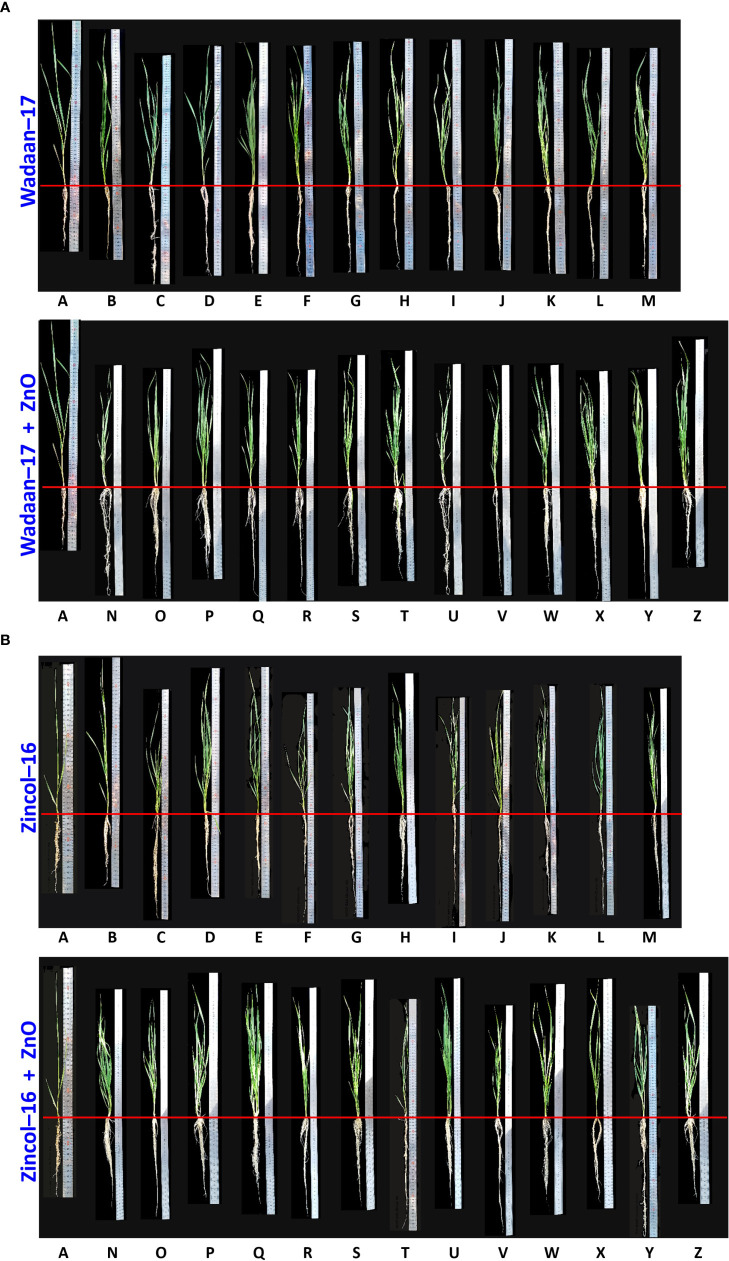
Shoot and root length of wheat varieties **(A)** Wadaan -17 and **(B)** Zincol-16 affected by inoculated ZSB with and without ZnO.Alphabets represent different treatments combinations. **^A^** Control; **^B^** NCCP-436; **^C^** NCCP-525; **^D^** NCCP-607; **^E^** NCCP-622; **^F^** NCCP-623; **^G^** NCCP-644; **^H^** NCCP-650; **^I^** NCCP-668; **^J^** NCCP-673; **^K^** NCCP-675; **^L^** NCCP-680; **^M^** Consortium; **^N^** NCCP-436+ZnO; **°**NCCP-525+ZnO; **^P^** NCCP-607+ZnO; **^Q^** NCCP-622+ZnO; **^R^** NCCP-623+ZnO; **^S^** NCCP-644+ZnO; **^T^** NCCP-650+ZnO; **^U^** NCCP-668+ZnO; **^V^** NCCP-673+ZnO; **^W^** NCCP-675+ZnO;**^X^** NCCP-680+ZnO; **^Y^** Consortium + ZnO and **^Z^** Insoluble Zn.

**Growth Parameters:** The Zn solubilization potential of the selected Zn-solubilizing bacteria was evaluated under glasshouse condition. In the present experiment, the selected bacterial strains were inoculated both with and without ZnO-treated sand. The results revealed that the inoculation of the selected ZSB strains (*Pantoea* sp. NCCP-525*, Klebsiella* sp. NCCP-607, *Klebsiella* sp. NCCP-623*, Brevibacterium* sp. NCCP-622, *Acinetobacter* sp. NCCP-644, *Acinetobacter* sp. NCCP-680, *Alcaligenes* sp. NCCP-650, *Citrobacter* sp. NCCP-668, *Exiguobacterium* sp. NCCP-673, and *Raoultella* sp. NCCP-675) produced encouraging effects. Kamran et al. (2017) reported that bacteria, i.e., *Pseudomonas fragi*, *Pantoea dispersa*, *Pantoea agglomerans*, *E. cloacae*, and *Rhizobium* spp. solubilized Zn and enhanced plant growth under sand culture. The wheat shoot, root, and Zn solubilization were enhanced through the inoculation of the selected strains. Most of the strains’ effects on wheat growth and Zn solubility are reported for the first time in this study. The results revealed that the inoculation of the selected ZSB along with ZnO (0.005% Zn) indicated a lessening in the shoot length with a positive impact of enormous root length and Zn concentration in the shoot (**Table 4** and **Figure 9**). These results are in line with those of Islam et al. (2014), who concluded that inoculation of *Pseudomonas aeruginosa* as PGPR along with Zn caused a reduction in the shoot length of wheat seedling, and a similar finding had also been observed by Jain et al. (2010) in sugarcane and Vivas et al. (2006) in *Trifolium repens*. However, contradictory observations regarding the shoot fresh weight, shoot dry weight, and shoot length were also reported in the findings of Kamran et al. (2017), in which different strains and Zn concentrations were applied. The probable reason of the impaired shoot length might be due to the higher Zn accumulation in the shoot, and an earlier report by Wei et al. (2022) stated that above the critical level of Zn content, plants eventually showed a falling tendency in shoot growth. The hermetic impact on wheat caused by high Zn exposure in another likely cause.

**Zn content in shoot:** The inoculation of the ZSB with and without ZnO enhanced the shoot Zn content, but the inoculation of *Pseudomonas* sp. NCCP-436 did not enhance the shoot Zn content more than the control. The inoculation of the ZSB strains in consortium with ZnO enhanced the Zn content in wheat shoot, and the conceivable reason might be due to the solubilization of Zn from ZnO. The observations of present investigation are corroborated by the findings of Kamran et al. (2017), who documented that the inoculation of different bacterial strains increased Zn solubilization from ZnCO_3_ and ultimately enhanced the shoot Zn content of wheat.

## Conclusion

The results of the present investigation demonstrate that indigenous novel bacterial species (*Pantoea* sp. NCCP-525, *Klebsiella* sp. NCCP-607, *Brevibacterium* sp. NCCP-622, *Klebsiella* sp. NCCP-623, *Acinetobacter* sp. NCCP-644, *Alcaligenes* sp. NCCP-650, *Citrobacter* sp. NCCP-668, *Exiguobacterium* sp. NCCP-673, *Raoultella* sp. NCCP-675, and *Acinetobacter* sp. NCCP-680) have the ability to solubilize insoluble sources of Zn and improve the growth of wheat. These bacterial strains have multifarious plant growth-promoting traits, including P solubilization and the presence of the *nifH* and *acdS* genes. Subsequently, these multi-trait bacterial strains can be attractive bio-inoculants for growth and to combat Zn deficiency in plants, where chemical Zn fertilizers are not cost-effective. In addition, the glasshouse experiment under sand culture recorded that 0.005% (50 mg kg^-1^) Zn from ZnO had no negative effect on wheat growth, while a greater concentration diminished the plant growth. Based on our results, we strongly recommend that researchers embark on further studies on the genetic and molecular mechanisms of Zn solubilization and further evaluation of these promising strains in field conditions to confirm their ability in the Zn biofortification of cereals.

## Data availability statement

The original contributions presented in the study are included in the article/**Supplementary Materials**. 16S rRNA Accession numbers of the strains are mentioned in the **Supplementary Materials**. Further inquiries can be directed to the corresponding author.

## Author contributions

IA, MZ and MS conceptualized and supervised the whole research study. MA, HT. and IA conducted the experiments, data analysis, and first draft manuscript writing. IA, SA and AM conducted the provision of the resources, molecular analysis, and provided the basic lab facilities. All authors contributed to the article and approved the submitted version.
