# Supplementary material for: Growth improvement of wheat (Triticum aestivum) and zinc biofortification using potent zinc-solubilizing bacteria
Source: Front Plant Sci. 2023 May 12;14:1140454. doi: 10.3389/fpls.2023.1140454 (PMC10213544; doi:10.3389/fpls.2023.1140454)
Supplement: Supplementary file 1 [file Table_1.docx]

**Growth Improvement of Wheat (Triticum aestivum) and Zinc Biofortification using Potent Zinc Solubilizing Bacteria**

Murad Ali ^1, 2, 3^, Iftikhar Ahmed^1 *^, Hamza Tariq^1^, Saira Abbas^4^, Munir Hussain Zia^5^, Amer Mumtaz^6^, Muhammad Sharif^2^

^1^ National Culture Collection of Pakistan (NCCP), Land Resources Research Institute (LRRI), National Agricultural Research Centre (NARC), Park Road, Islamabad, Pakistan

^2^ Department of Soil and Environmental Sciences, The University of Agriculture, Peshawar, Pakistan

^3^ Cereal Crops Research Institute (CCRI), Pirsabak, Nowshera, Pakistan

^4^ Department of Zoology, University of Science and Technology, Bannu, Pakistan

^5^ Research and Development Coordination, Fauji Fertilizer Company (FFC), Rawalpindi, Pakistan

^6^ Food Sciences Research Institute (FSRI), National Agricultural Research Centre (NARC), Park Road, Islamabad, Pakistan

*Corresponding author:

**Iftikhar Ahmed**

Email: iftikhar.ahmed@parc.gov.pk

**Supplementary Table 1.** Bacterial strains used in this study, were previously identified based on 16S rRNA gene sequencing. These strains were collected from NCCP to screen for Zn solubility potential.

| **S. NO** | **Strain I.D.** | **Accession No.** | **Nucleotide length (bp)** | **Closely related taxa** | **Similarity (%)** | **Number of strains having similar >97 (>98) %** | **Image** |
| --- | --- | --- | --- | --- | --- | --- | --- |
|  | NCCP-11 | AB618145 | 1421 | *Cellulomonas pakistanensis* JCM 18755^T^ (BBHV01000063) | 100 | 3(3) | *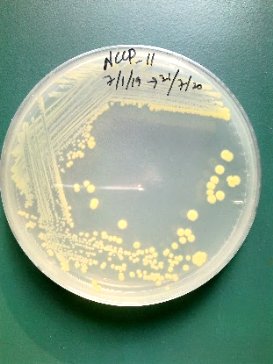* |
|  | NCCP-18 | AB576763 | 1491 | *Pseudomonas paralactis* DSM 29164^T^ (KP756921) | 100 | 51(51) | 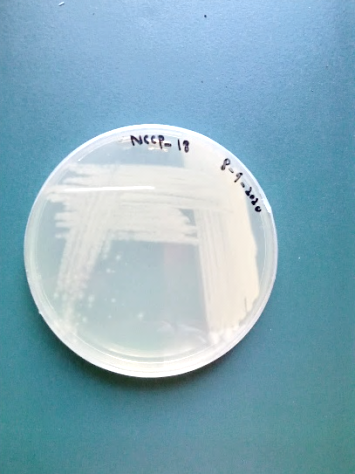 |
|  | NCCP-28 | AB547220 | 1430 | *Bacillus nealsonii* DSM 15077^T^(EU656111) | 97.97 | 6(0) | 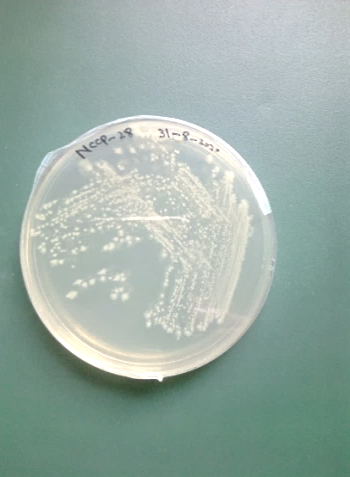 |
|  | NCCP-30 | AB547221 | 1437 | *Lysinibacillus macroides* DSM 54^T^ (LGCI01000008) | 99.37 | 14(10) | 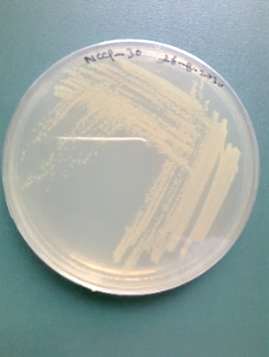 |
|  | NCCP-48 | AB547228 | 1446 | *Bacillus velezensis* CR-502^T^ (AY603658) | 99.64 | 24(17) | 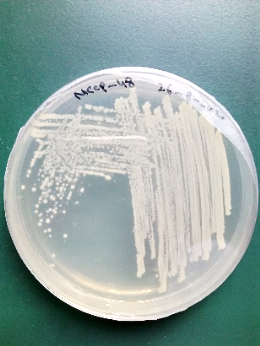 |
|  | NCCP-49 | AB547229 | 1446 | *Bacillus siamensis* KCTC 13613^T^ (AJVF01000043) | 99.44 | 23(15) | 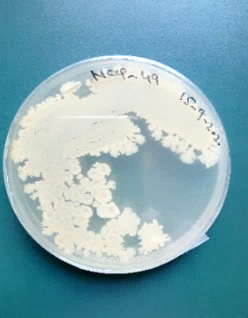 |
|  | NCCP-50 | AB547230 | 1438 | *Bacillus halotolerans* ATCC 25096^T^(LPVF01000003) | 99.58 | 23(17) | 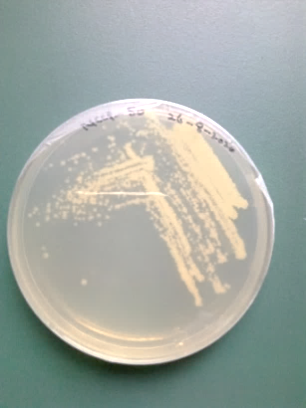 |
|  | NCCP-51 | AB665172 | 1443 | *Bacillus subtilis subsp. stercoris* D7XPN1^T^(JHCA01000027) | 99.36 | 22(15) | 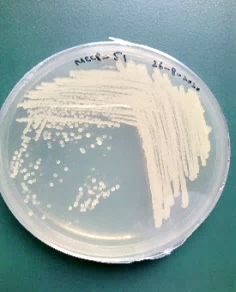 |
|  | NCCP-54 | AB558495 | 1481 | *Lysinibacillus pakistanensis* JCM18776^T^  (BBDJ01000063) | 99.59 | 11(8) | 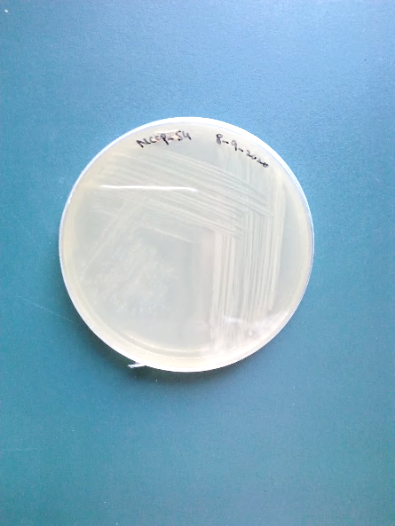 |
|  | NCCP-142 | AB548822 | 1465 | *Klebsiella pneumoniae subsp. ozaenae*  ATCC 11296^T^ Y17654 | 99.65 | 50(21) | 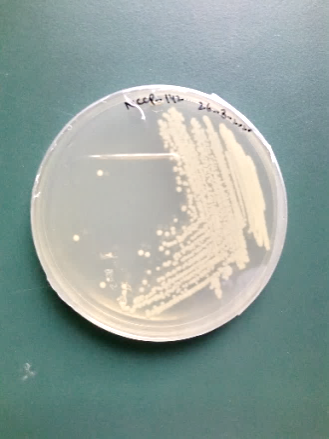 |
|  | NCCP-147 | AB549434 | 1432 | *Brevundimonas vesicularis* NBRC 12165^T^ (BCWM01000033) | 99.20 | 17(5) | *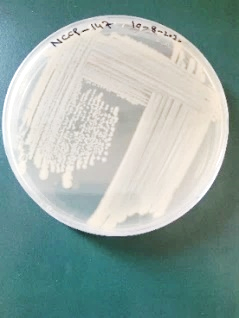* |
|  | NCCP-195 | AB662961 | 1419 | *Klebsiella aerogenes* KCTC 2190^T^ (CP002824) | 98.45 | 46(3) | 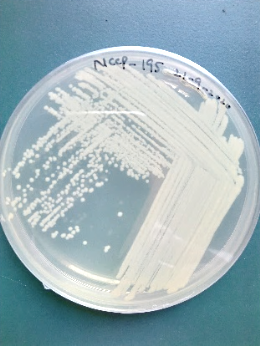 |
|  | NCCP-200 | AB662965 | 1476 | *Serratia glossinae* C1^T^ (FJ790328) | 98.63 | 8(2) | 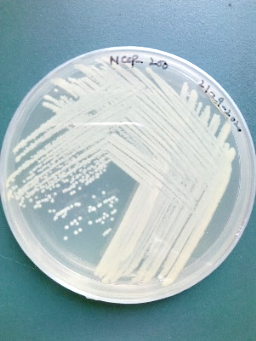 |
|  | NCCP-231 | AB610883 | 1462 | *Kosakonia oryzae* Ola 51^T^ (CP014007) | 99.93 | 50(16) | 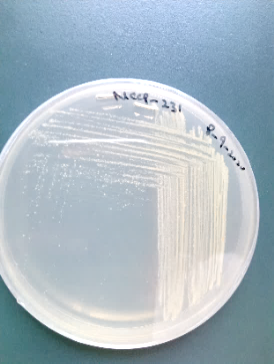 |
|  | NCCP-241 | AB665215 | 1438 | *Pantoea endophytica* 596T(PJRT01000022) | 99.72 | 50(20) | 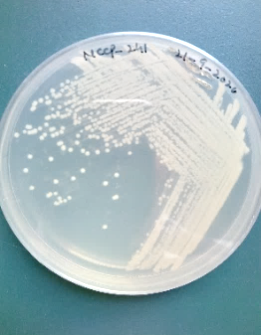 |
|  | NCCP-246 | AB610802 | 1,460 | *Sphingobacterium pakistanense* NCCP246^T^  (NR_113311) | 100 | 5(2) | 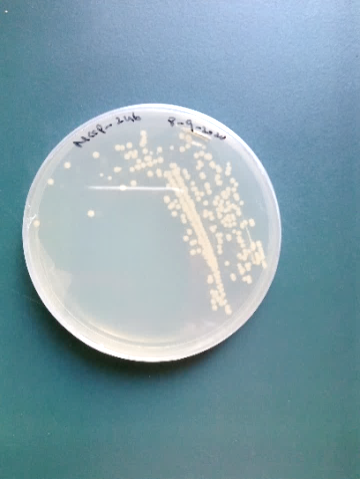 |
|  | NCCP-414 | LC488899 | 1407 | *Acinetobacter indicus* CIP 110367T(KI530754) | 99.50 | 1(1) | 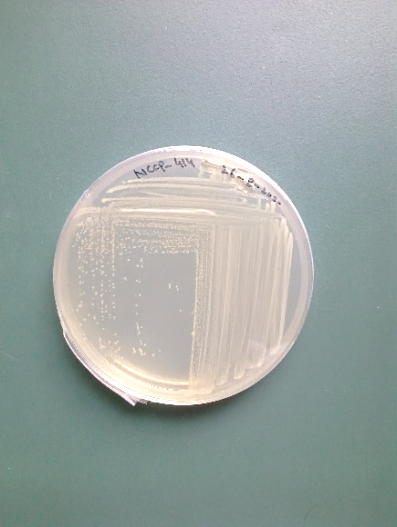 |
|  | NCCP-436 | LC488921 | 1409 | *Pseudomonas khazarica* TBZ2^T^ (KX712072) | 97.94 | 18(0) | 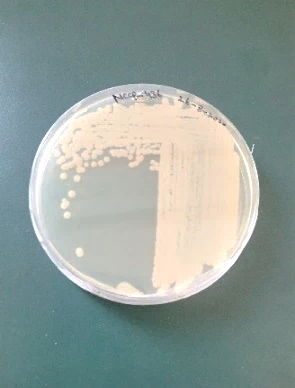 |
|  | NCCP-510 | AB740336 | 730 | *Pantoea dispersa* LMG 2603^T^ (DQ504305) | 97.76 | 0(3) | 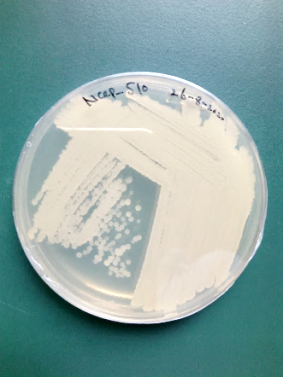 |
|  | NCCP-517 | AB740343 | 1431 | *Bacillus halotolerans* ATCC 25096^T^ (LPVF01000003) | 99.65 | 23(17) | 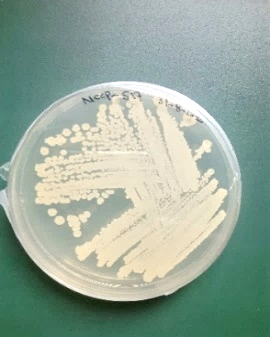 |
|  | NCCP-520 | AB740346 | 780 | *Curtobacterium citreum* DSM 20528^T^(X77436) | 99.62 | 8(8) | *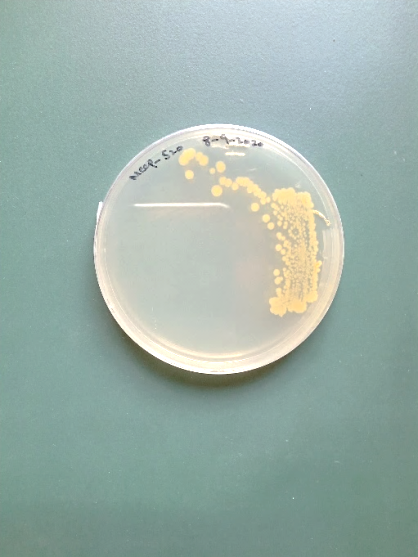* |
|  | NCCP-523 | AB740349 | 1401 | *Kocuria palustris* DSM 11925^T^(Y16263) | 99.28 | 2(2) | 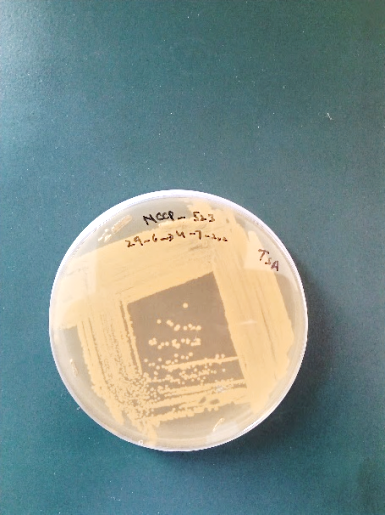 |
|  | NCCP-525 | AB740351 | 661 | *Pantoea dispersa* LMG 2603^T^(DQ504305) | 99.85 | 26(4) | 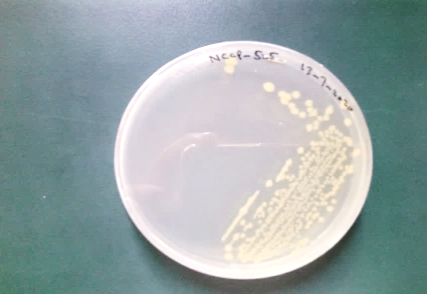 |
|  | NCCP-601 | AB920786 | 1416 | *Staphylococcus xylosus* CCM 2738^T^ (MRZO01000018) | 99.93 | 50(29) | 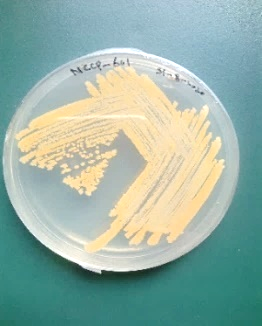 |
|  | NCCP-602 | AB920787 | 1432 | *Brevibacterium ammoniilyticum* A1^T^(JF937067) | 98.99 | 16(4) | 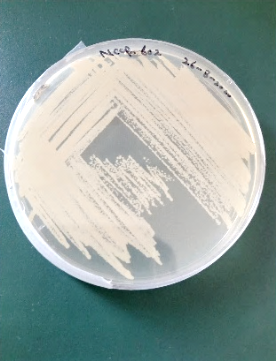 |
|  | NCCP-605 | AB920790 | 1393 | *Citrobacter murliniae* CDC 2970-59^T^(AF025369) | 99.93 | 50(50) | 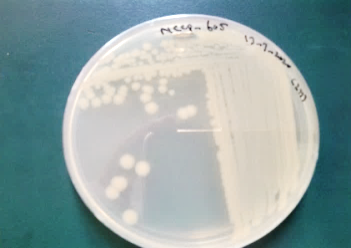 |
|  | NCCP-607 | AB920792 | 1427 | *Klebsiella aerogenes* KCTC 2190^T^ (CP002824) | 99.15 | 50(48) | 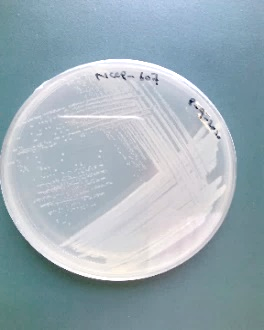 |
|  | NCCP-614 | AB920799 | 874 | *Stenotrophomonas bentonitica*BII-R7^T^(LT622838) | 100 | 50(4) | 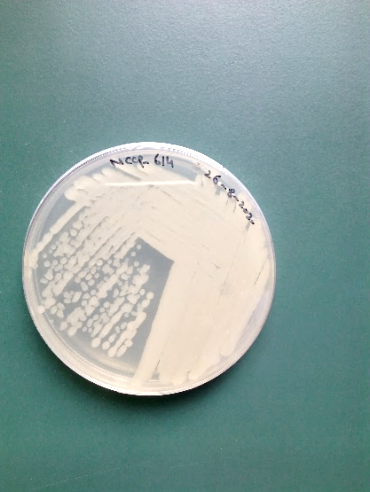 |
|  | NCCP-616 | AB920801 | 1368 | *Alcaligenes aquatilis* LMG 22996^T^(JX986974) | 100 | 7(7) | 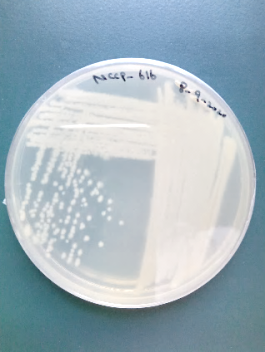 |
|  | NCCP-619 | AB920804 | 968 | *Mesobacillus persicus* B48^T^(HQ433471) | 97.52 | 2(0) | 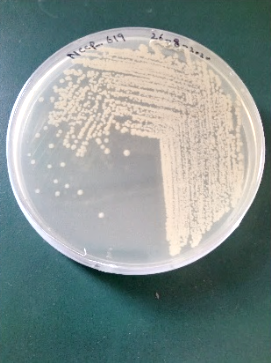 |
|  | NCCP-621 | AB920806 | 852 | *Exiguobacterium indicum*HHS31^T^(AJ846291) | 100 | 10(6) | *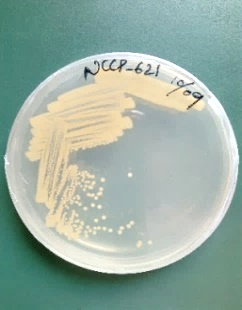* |
|  | NCCP-622 | AB920807 | 1399 | *Brevibacterium frigoritolerans* DSM 8801^T^(AM747813) | 99.79 | 12(4) | 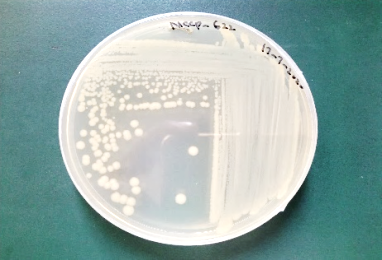 |
|  | NCCP-623 | AB920808 | 815 | *Klebsiella pneumoniae subsp. Pneumonia* DSM 30104^T^ (AJJI01000018) | 99.75 | 50(50) | 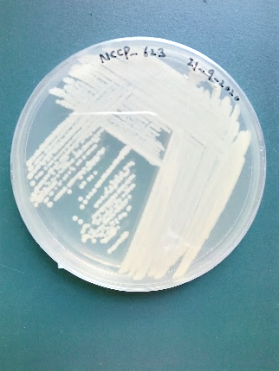 |
|  | NCCP-628 | AB920810 | 1132 | *Staphylococcus equorum subsp. Equorum* ATCC 43958^T^ (AB009939) | 99.73 | 25(15) | 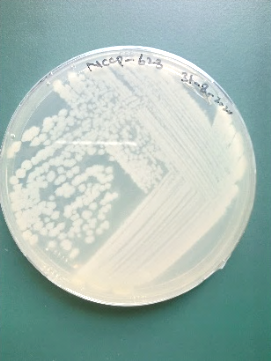 |
|  | NCCP-631 | AB920812 | 861 | *Klebsiella pneumonia subsp. rhinoscleromatis* ATCC13884^T^ (ACZD01000038) | 99.77 | 50(50) | 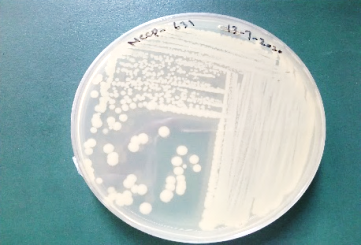 |
|  | NCCP-632 | AB920813 | 946 | *Pseudomonas aeruginosa* JCM 5962^T^ (BAMA01000316) | 99.68 | 9(2) | 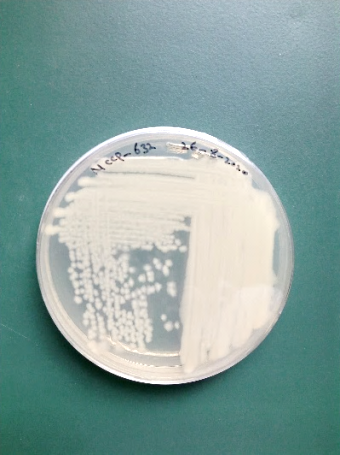 |
|  | NCCP-638 | AB920819 | 965 | *Bacillus flexus* NBRC 15715^T^ (BCVD01000224) | 100 | 6(5) | 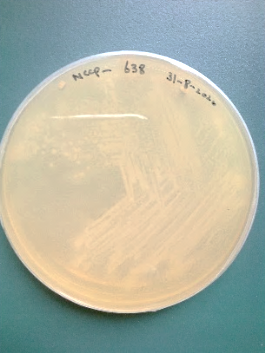 |
|  | NCCP-644 | AB916465 | 1413 | *Acinetobacter bohemicus* ANC 3994^T^(KB849175) | 99.72 | 37(6) | 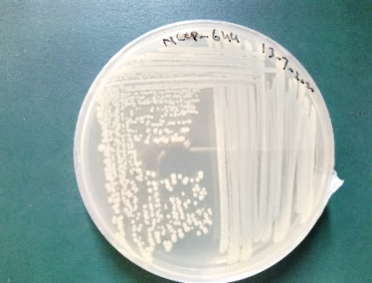 |
|  | NCCP-645 | AB920823 | 966 | *Pseudomonas bubulae* TH39^T^(KX186949) | 99.90 | 50(50) | 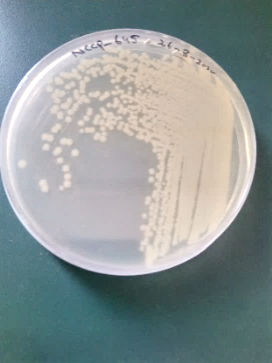 |
|  | NCCP-646 | AB920824 | 840 | *Pseudomonas lactis* DSM 29167^T^ (JYLO01000038) | 98.33 | 17(6) | 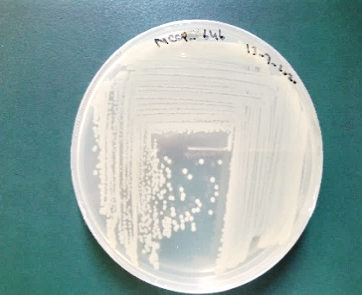 |
|  | NCCP-648 | AB920826 | 1390 | *Psychrobacter faecalis* Iso-46^T^(AJ42152 | 99.78 | 33(9) | 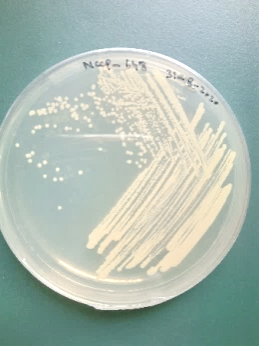 |
|  | NCCP-650 | AB920828 | 1412 | *Alcaligenes pakistanensis* NCCP-650^T^ (AB920828) | 100 | 10(6) | 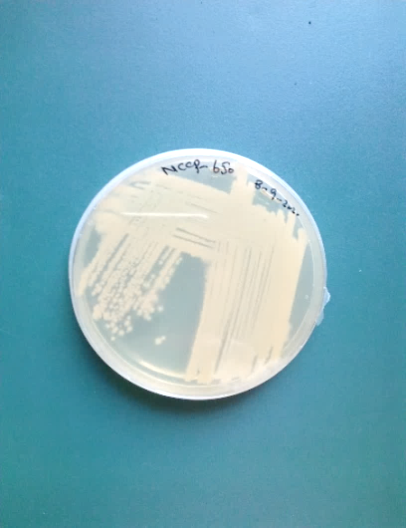 |
|  | NCCP-651 | AB920829 | 1403 | *Bacillus cereus* ATCC 14579^T^(AE016877) | 100 | 37(36) | 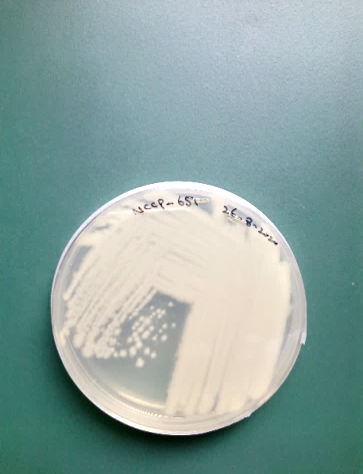 |
|  | NCCP-652 | AB920830 | 846 | *Thauera mechernichensis* TL1^T^(Y17590) | 99.05 | 6(6) | 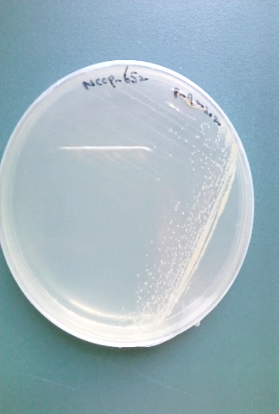 |
|  | NCCP-654 | AB920832 | 1118 | *Pseudomonas extremaustralis* 14-3^T^(AHIP01000073) | 99.19 | 53(53) | 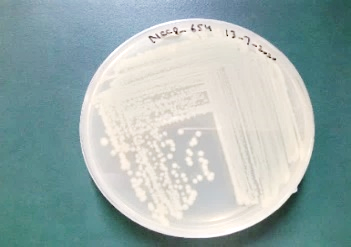 |
|  | NCCP-660 | AB920838 | 881 | *Staphylococcus haemolyticus* MTCC3383^T^  (LILF01000056) | 100 | 49(21) | 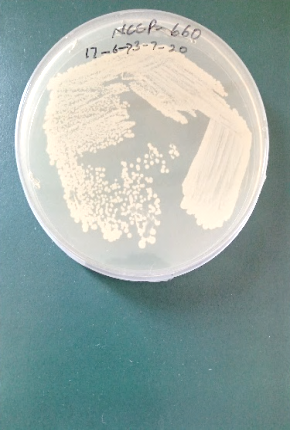 |
|  | NCCP-661 | AB920839 | 1092 | *Brachybacterium nesterenkovii* CIP104813^T^  (FWFG01000034) | 99.18 | 7(2) | 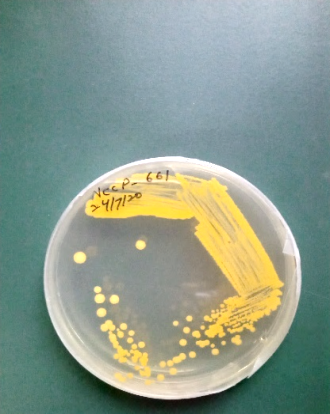 |
|  | NCCP-662 | AB968093 | 1492 | *Metabacillus malikii* NCCP-662^T^(AB968093) | 100 | 6(3) | *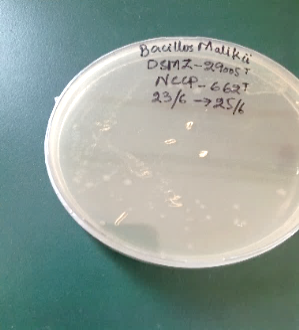* |
|  | NCCP-663 | AB968094 | 858 | *Alcaligenes faecalis subsp. phenolicus* DSM 16503^T^ (AUBT01000026) | 99.77 | 7(6) | 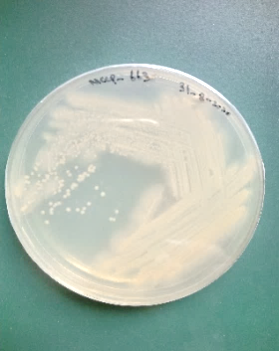 |
|  | NCCP-666 | AB968095 | 1494 | *Bacillus cohnii* NBRC 15565^T^ (BCUW01000190) | 100 | 10(4) | 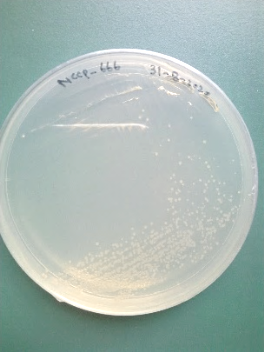 |
|  | NCCP-667 | AB968096 | 1479 | *Alcaligenes pakistanensis* NCCP-650^T^ (AB920828) | 100 | 7(6) | 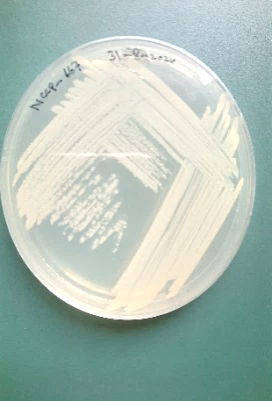 |
|  | NCCP-668 | AB968097 | 829 | *Citrobacter amalonaticus* CECT 863^T^(FR870441) | 100 | 44(12) | 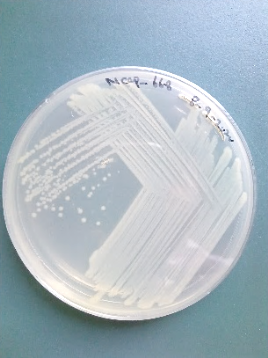 |
|  | NCCP-670 | AB968098 | 811 | *Brevundimonas mediterranea* V4.BO.10^T^ (AJ227801) | 99.88 | 11(5) | 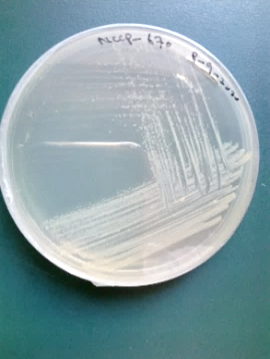 |
|  | NCCP-671 | AB968099 | 882 | *Bacillus paralicheniformis* KJ-16^T^(KY694465) | 99.89 | 23(18) | 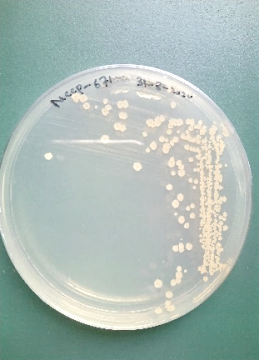 |
|  | NCCP-673 | AB968101 | 892 | *Exiguobacterium mexicanum*8N^T^ (AM072764) | 99.78 | 8(5) | 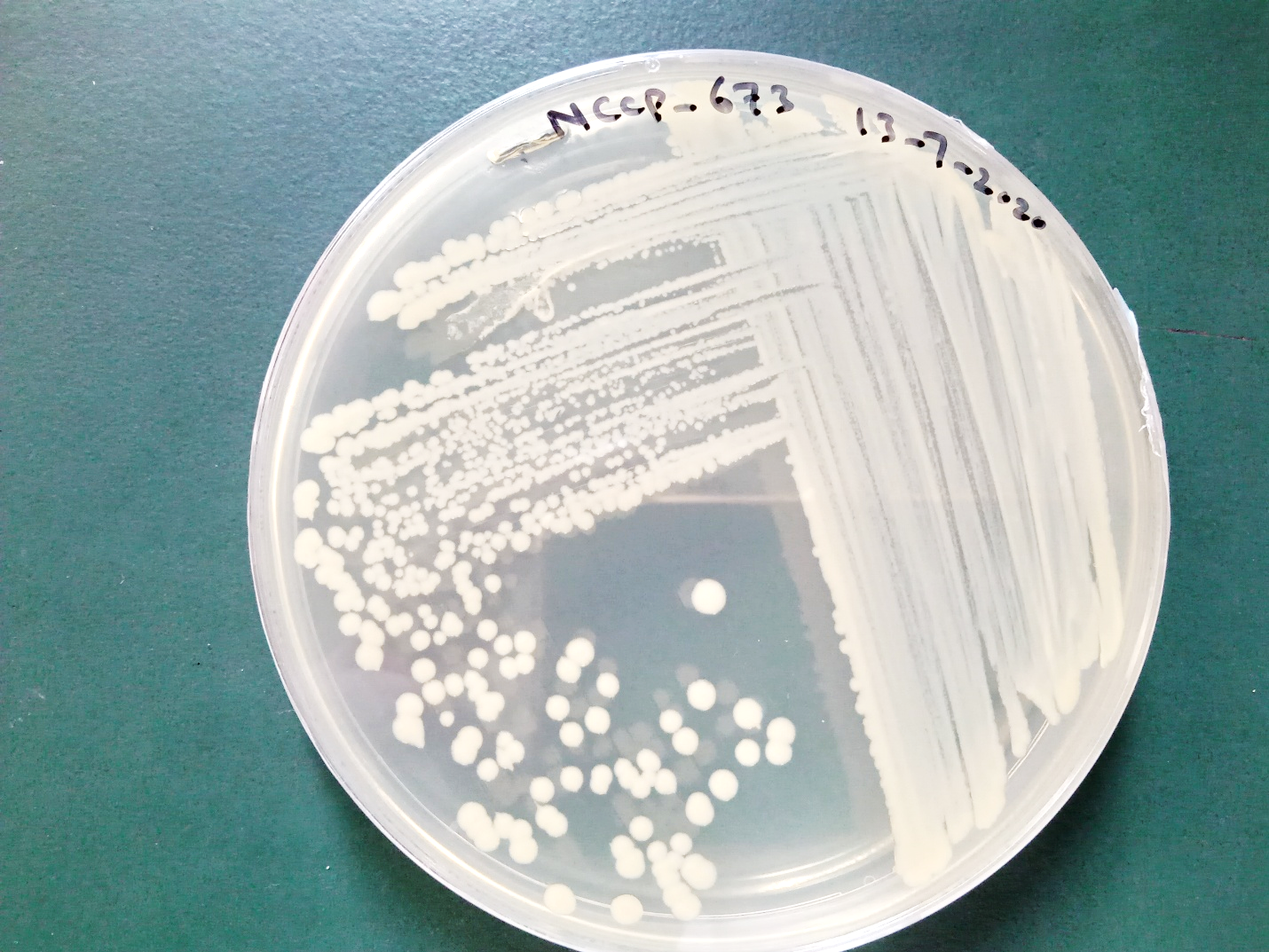 |
|  | NCCP-675 | AB968102 | 868 | *Raoultella ornithinolytica* JCM 6096^T^(AJ251467) | 100 | 50(34) | 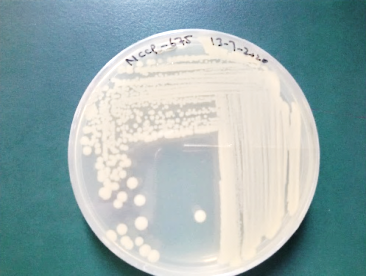 |
|  | NCCP-677 | AB968103 | 1492 | *Paenibacillus motobuensis*MC10^T^(AY741810) | 99.86 | 3(3) | 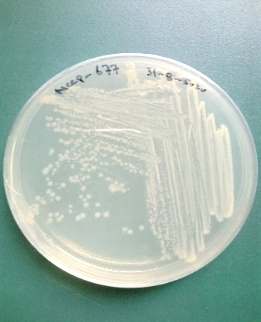 |
|  | NCCP-680 | AB968105 | 1501 | *Acinetobacter bouvetii* DSM 14964^T^ (APQD01000004) | 100 | 50(16) | 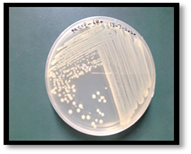 |
|  | NCCP-802 | CP023481 | 4659467 | *Bacillus glycinifermentan* | 100 | 1(1) | 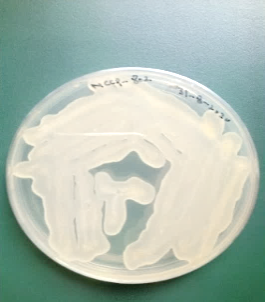 |
|  | NCCP-905 | AB970648 | 944 | *Planococcus citreus* DSM 20549^T^(RCCP01000013) | 100 | 29(12) | 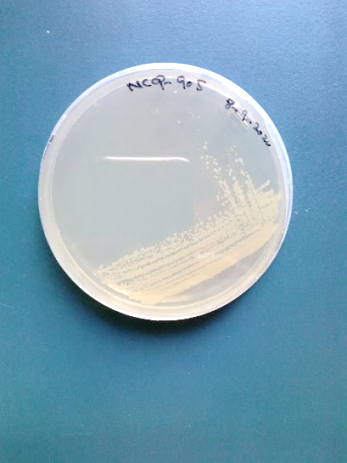 |
|  | NCCP-925 | AB970666 | 1420 | *Bacillus paralicheniformis*KJ-16^T^(KY694465) | 99.22 | 23(8) | 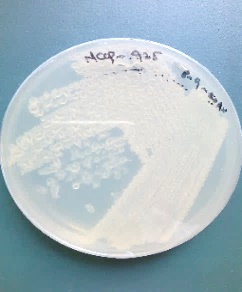 |
|  | NCCP-936 | AB970677 | 1128 | *Brachybacterium phenoliresistens* phenol-A^T^(DQ822566) | 99.65 | 2(1) | 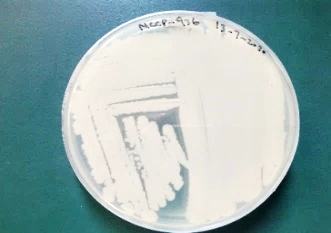 |
|  | NCCP-953 | AB970688 | 970 | *Staphylococcus equorum* subsp. *equorum* ATCC 43958^T^ (AB009939) | 100 | 35(17) | 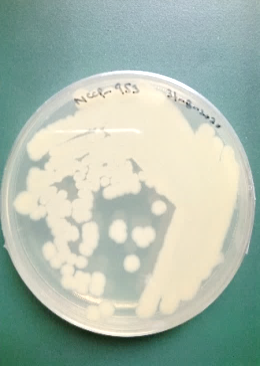 |
|  | NCCP-958 | AB970692 | 1130 | *Staphylococcus saprophyticus* subsp. *Saprophyticus* ATCC 15305^T^ (AP008934) | 100 | 46(23) | 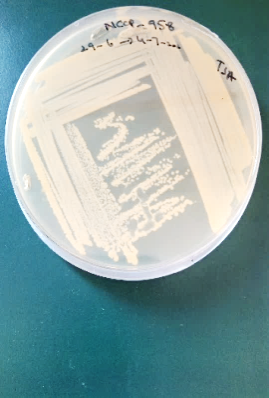 |
|  | NCCP-959 | AB970693 | 981 | *Oceanobacillus oncorhynchi* subsp. *Incaldanensis*20AG^T^ (AJ640134) | 99.80 | 9(6) | 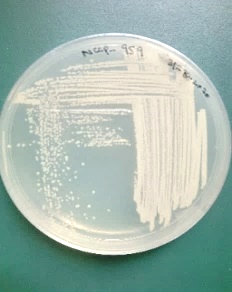 |
|  | NCCP-960 | AB970694 | 983 | *Staphylococcus sciuri* DSM 20345^T^ (AJ421446) | 99.39 | 5(5) | 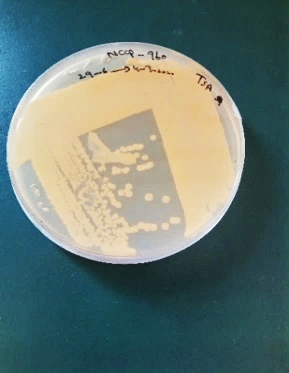 |
|  | NCCP-969 | AB970696 | 660 | *Bacillus marisflavi JCM* 11544^T^(LGUE01000011) | 98.79 | 10(4) | 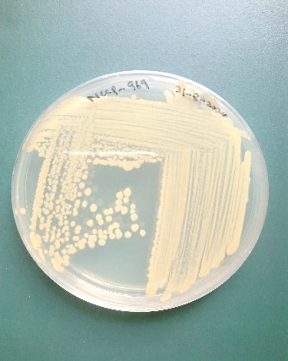 |
|  | NCCP-979 | AB970704 | 1119 | *Bacillus marisflavi* JCM 11544^T^ (LGUE01000011) | 99.55 | 8(4) | 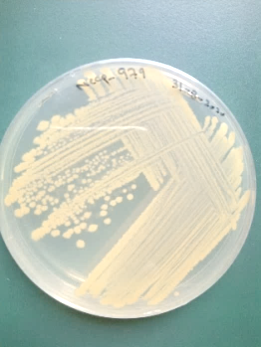 |
|  | NCCP-2050 | LC710227 | 1448 | *Plannococcus* sp. (NR_144714.1) | 98.27 | 27(5) | *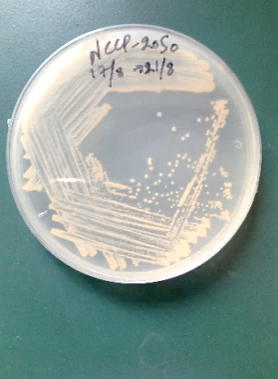* |
