# Supplementary material for: Growth improvement of wheat (Triticum aestivum) and zinc biofortification using potent zinc-solubilizing bacteria
Source: Front Plant Sci. 2023 May 12;14:1140454. doi: 10.3389/fpls.2023.1140454 (PMC10213544; doi:10.3389/fpls.2023.1140454)
Supplement: Supplementary file 2 [file Table_2.docx]

**Growth Improvement of Wheat (Triticum aestivum) and Zinc Biofortification using Potent Zinc Solubilizing Bacteria**

Murad Ali ^1, 2, 3^, Iftikhar Ahmed^1 *^, Hamza Tariq^1^, Saira Abbas^4^, Munir Hussain Zia^5^, Amer Mumtaz^6^, Muhammad Sharif^2^

^1^ National Culture Collection of Pakistan (NCCP), Land Resources Research Institute (LRRI), National Agricultural Research Centre (NARC), Park Road, Islamabad, Pakistan

^2^ Department of Soil and Environmental Sciences, The University of Agriculture, Peshawar, Pakistan

^3^ Cereal Crops Research Institute (CCRI), Pirsabak, Nowshera, Pakistan

^4^ Department of Zoology, University of Science and Technology, Bannu, Pakistan

^5^ Research and Development Coordination, Fauji Fertilizer Company (FFC), Rawalpindi, Pakistan

^6^ Food Sciences Research Institute (FSRI), National Agricultural Research Centre (NARC), Park Road, Islamabad, Pakistan

*Corresponding author:

**Iftikhar Ahmed**

Email: iftikhar.ahmed@parc.gov.pk

**Supplementary Table 2.** Plant growth promoting (PGP) activities of Zn solubilizing strains

| S. No. | Strain I.D. | Solubilization of ZnO | Halo zone of ZnO solubilizing strain | Solubilization of ZnCO_3_ | Halo zone of ZnCO3 solubilizing strain | P solubilization | Halozone of P solubilization | Qualitative test of IAA | NaCl tolerance (%) |
| --- | --- | --- | --- | --- | --- | --- | --- | --- | --- |
|  | **NCCP-20** | **+** | **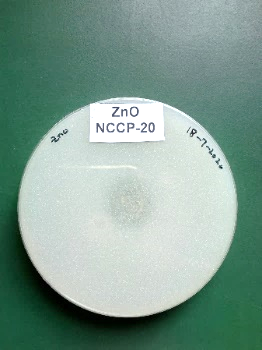** | **+** | **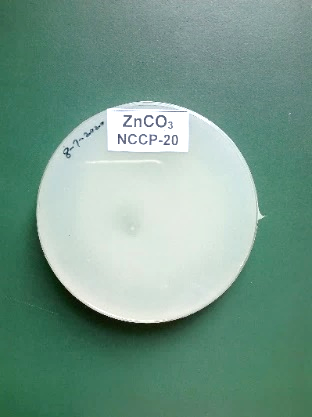** | **-** |  | **+** | 0-10 |
|  | **NCCP-27** | **++** | **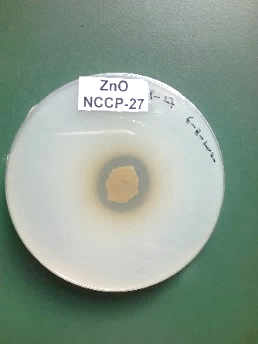** | **-** |  | **-** |  | **-** | 0-12 |
|  | **NCCP-46** | **+** | **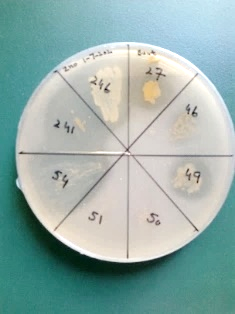** | **-** |  | **-** |  | **-** | 0-14 |
|  | **NCCP-49** | **+** | **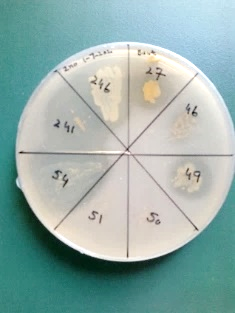** | **-** |  | **-** |  | **-** | 0-14 |
|  | **NCCP-54** | **+** | **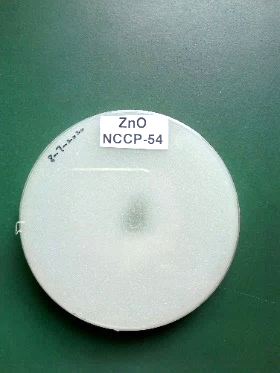** | **-** |  | **+++** | **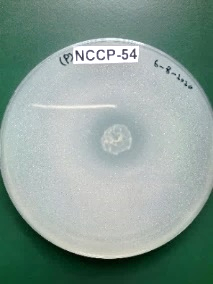** | **++** | 0-8 |
|  | **NCCP-147** | **++** | **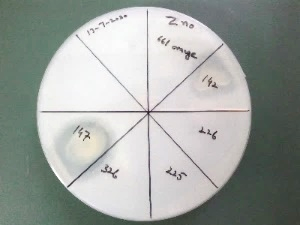** | **++** | **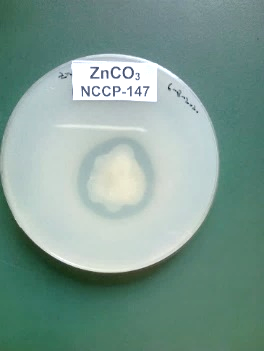** | **++** | **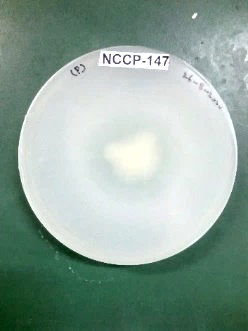** | **-** | 0-8 |
|  | **NCCP-195** | **+** | **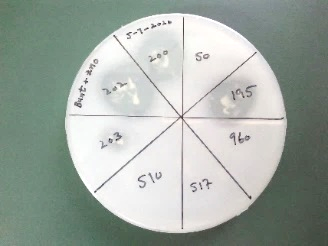** | **+** | **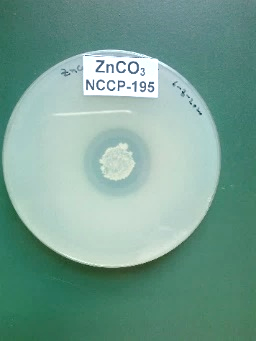** | **-** |  | **-** | 0-8 |
|  | **NCCP-200** | **+** | **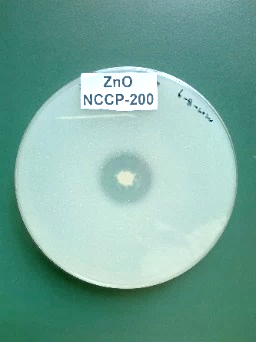** | **+** | **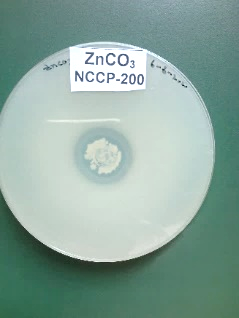** | **-** |  | **+** | 0-6 |
|  | **NCCP-202** | **+** | **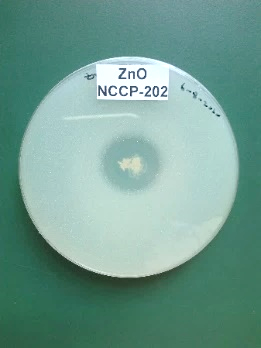** | **+** | **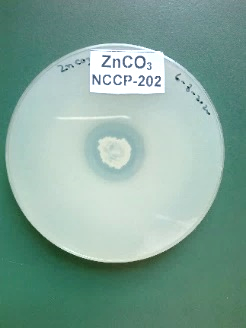** | **-** |  | **-** | 0-6 |
|  | **NCCP-241** | **+++** | **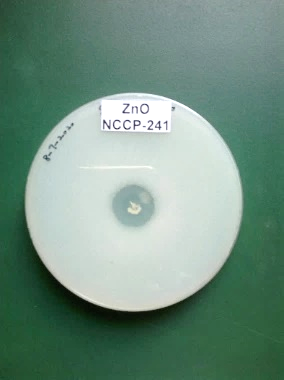** | **-** |  | **+++** | **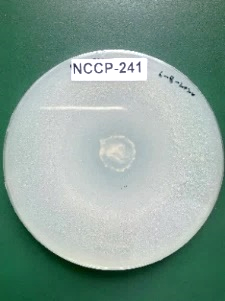** | **-** | 0-8 |
|  | **NCCP-246** | **++** | **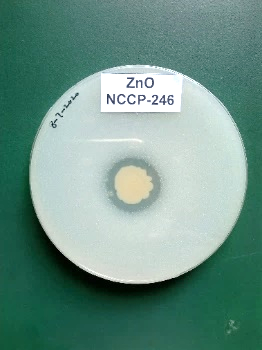** | **+** | **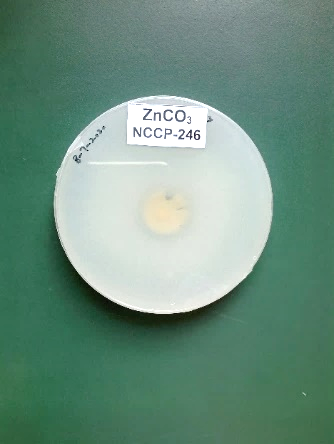** | **-** |  | **+** | 0-4 |
|  | **NCCP-493** | **+++** | **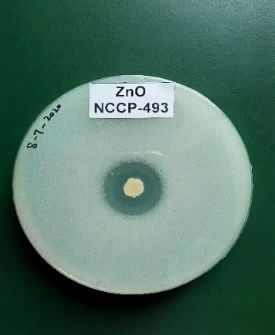** | **+** | **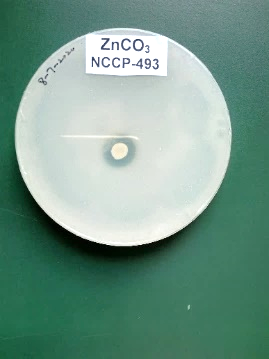** | **-** |  | **-** | 0-6 |
|  | **NCCP-522** | **++++** | **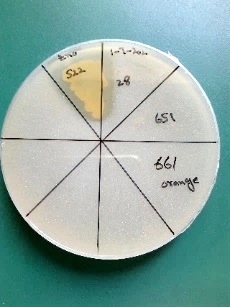** | **+++** | **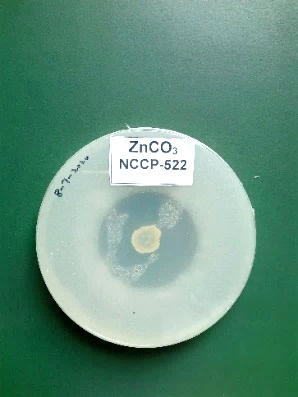** | **+** | **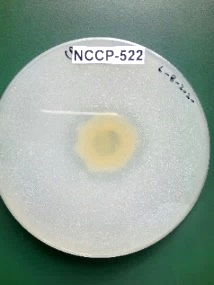** | **-** | 0-8 |
|  | **NCCP-525** | **++++** | **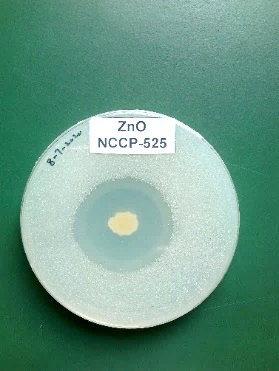** | **++++** | **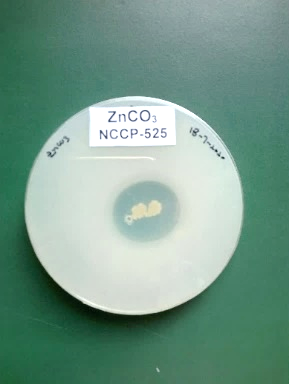** | **+++** | **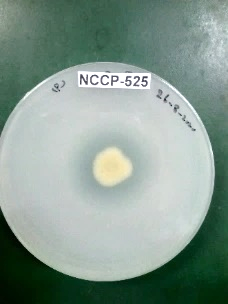** | **+** | 0-8 |
|  | **NCCP-605** | **++++** | **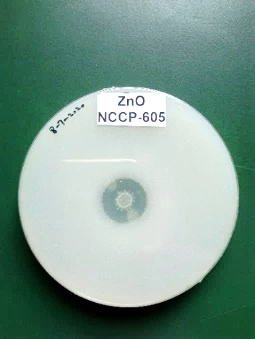** | **+++** | **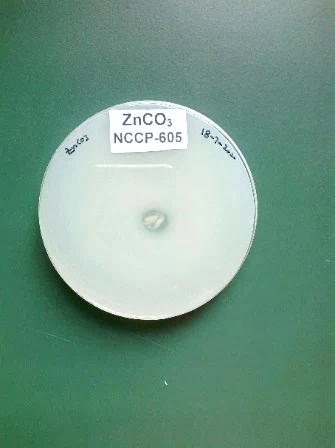** | **++** | **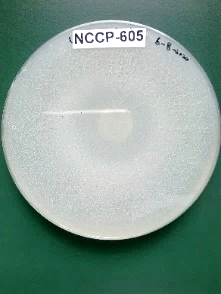** | **-** | 0-8 |
|  | **NCCP-607** | **++++** | **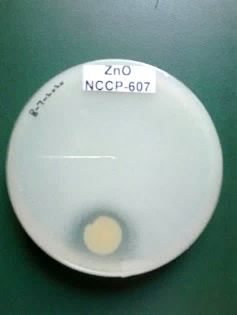** | **++++** | **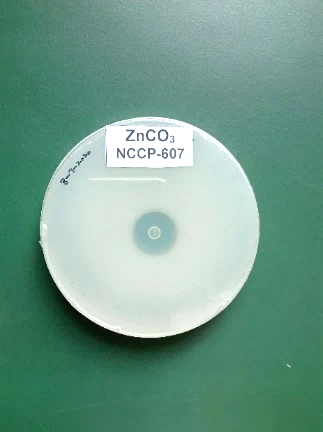** | **++++** | **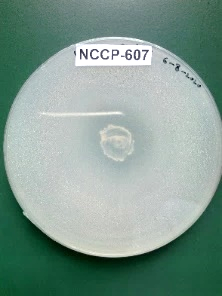** | **+** | 0-6 |
|  | **NCCP-616** | **++** | **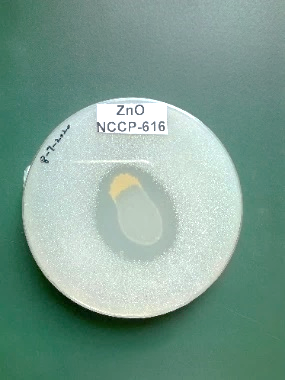** | **+** | **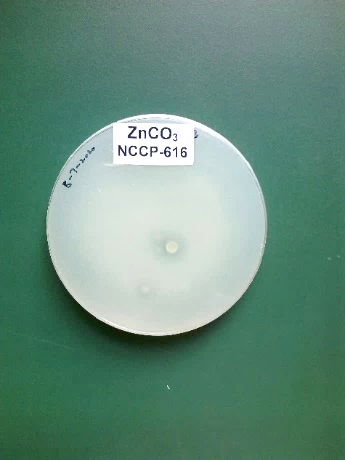** | **-** |  | **++** | 0-12 |
|  | **NCCP-622** | **++++** | **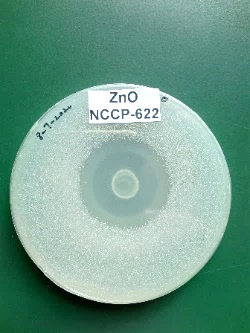** | **+++** | **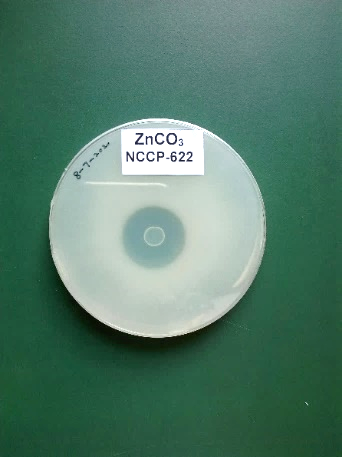** | **+++** | **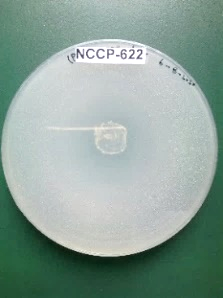** | **+** | 0-6 |
|  | **NCCP-623** | **++++** | **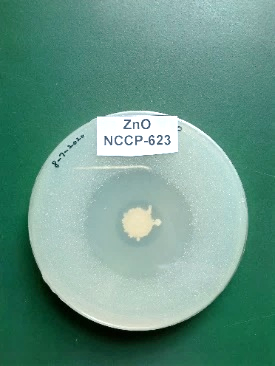** | **+++** | **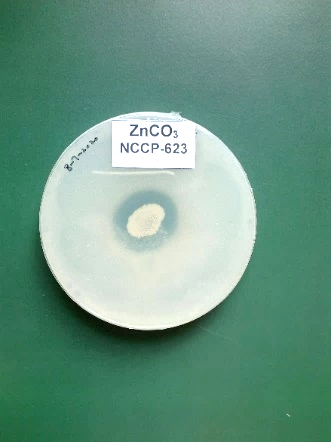** | **++++** | **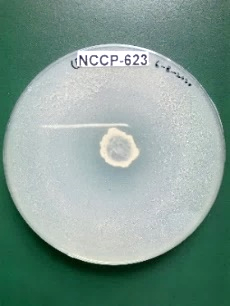** | **+** | 0-8 |
|  | **NCCP-628** | **+** | **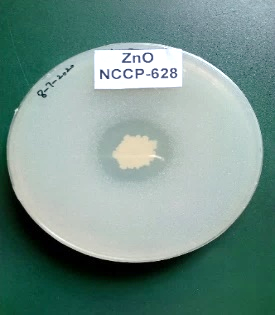** | **+** | **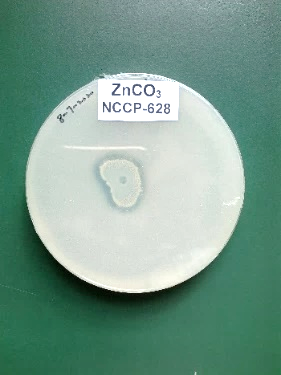** | **-** |  | **+** | 0-14 |
|  | **NCCP-631** | **+++** | **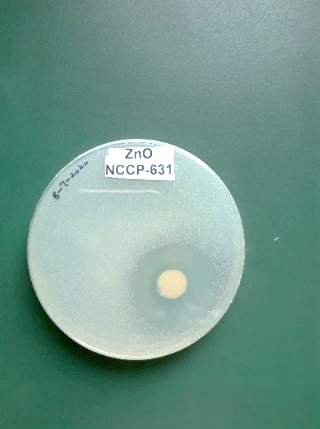** | **+** | **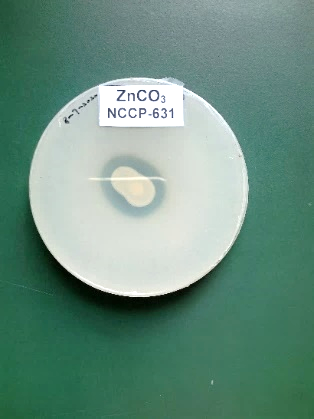** | **-** |  | **+** | 0-8 |
|  | **NCCP-644** | **++++** | **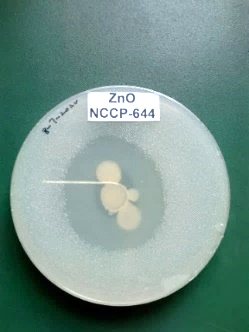** | **++++** | **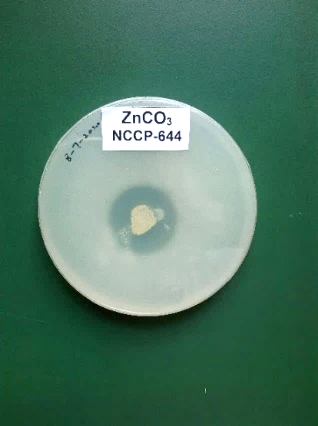** | **++++** | **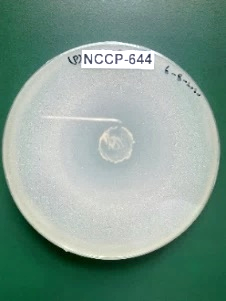** | **+** | 0-6 |
|  | **NCCP-646** | **+** | **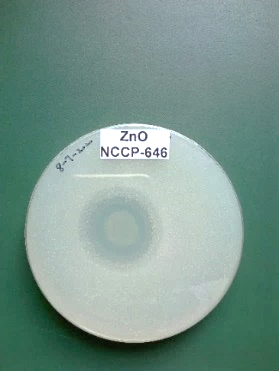** | **+** | **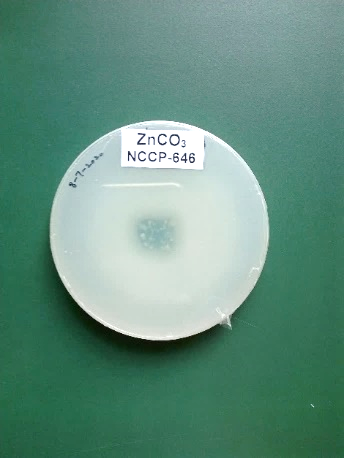** | **-** |  | **+** | 0-6 |
|  | **NCCP-650** | **+** | **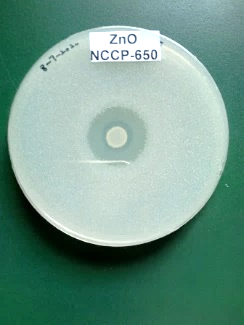** | **+** | **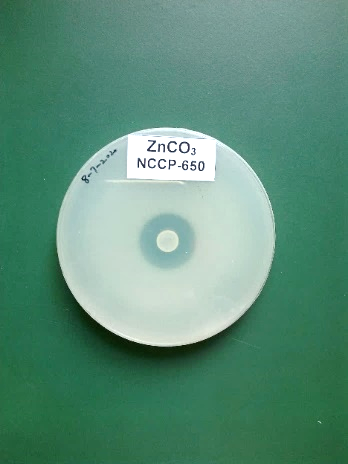** | **+** | **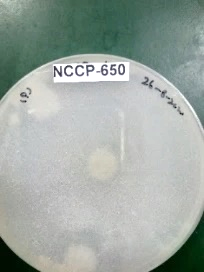** | **+** | 0-10 |
|  | **NCCP-654** | **+** | **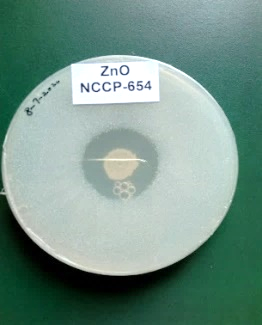** | **+** | **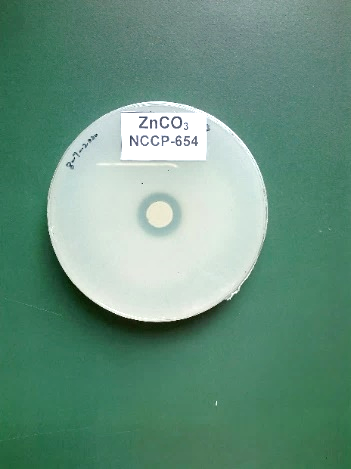** | **++** | **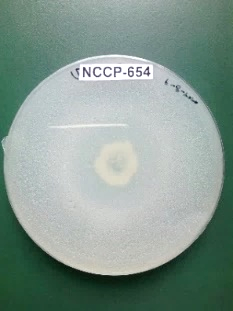** | **+** | 0-6 |
|  | **NCCP-668** | **++++** | **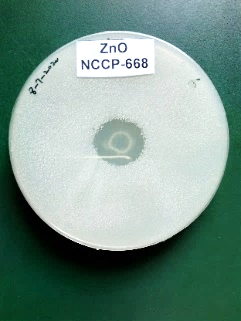** | **++++** | **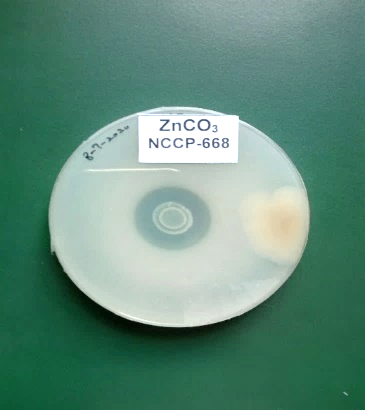** | **+++** | **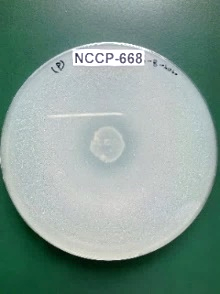** | **+++** | 0-8 |
|  | **NCCP-673** | **++++** | **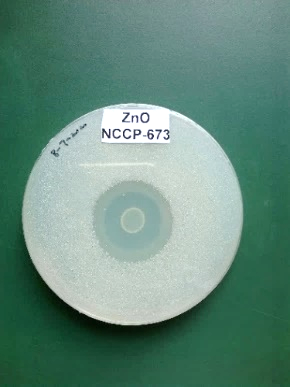** | **++++** | **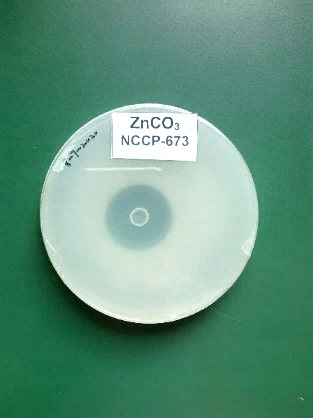** | **++++** | **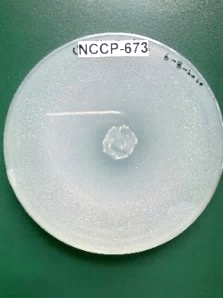** | **+** | 0-6 |
|  | **NCCP-675** | **++++** | **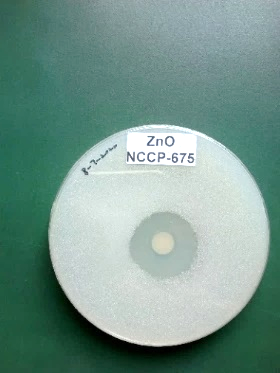** | **++++** | **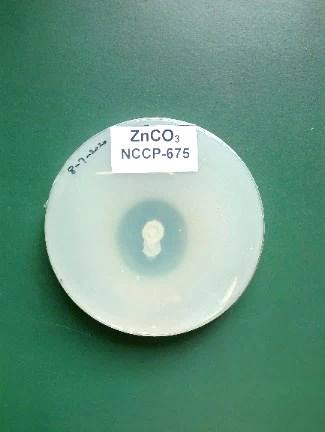** | **++++** | **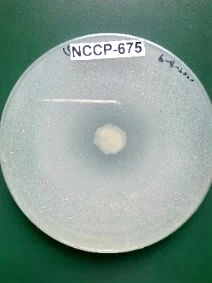** | **+++** | 0-6 |
|  | **NCCP-680** | **++++** | **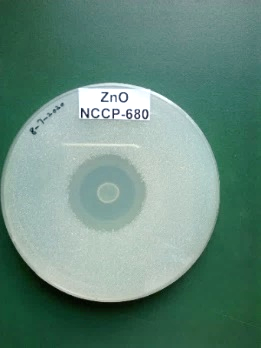** | **++++** | **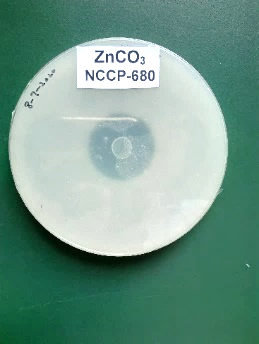** | **++++** | **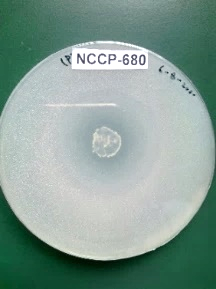** | **+** | 0-6 |
|  | **NCCP-936** | **+** | **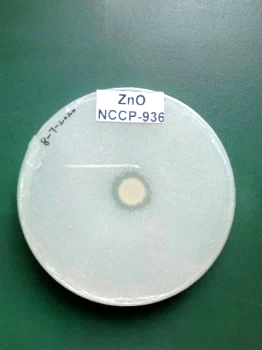** | **+** | **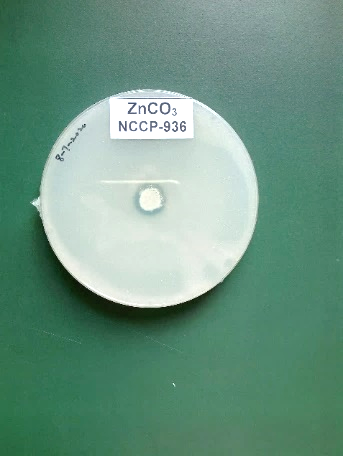** | **-** |  | **-** | 0-14 |

(-)= Negative; (+) = low; (++) = Moderate; (+++) = High; (++++) = Very high solubility.

* Initial screening of some bacterial strains for Zn solubilization was earlier published by Ali et al. (2022)
